# α-Acetoxyarone synthesis via iodine-catalyzed and *tert*-butyl hydroperoxide-mediateded self-intermolecular oxidative coupling of aryl ketones

**DOI:** 10.3762/bjoc.13.107

**Published:** 2017-06-06

**Authors:** Liquan Tan, Cui Chen, Weibing Liu

**Affiliations:** 1 College of Chemical Engineering, Guangdong University of Petrochemical Technology, 2 Guandu Road, Maoming 525000, P. R. China. Fax: +86-668-2923575; Tel: +86-668-2923444

**Keywords:** aryl ketones, iodine, self-intermolecular oxidative coupling, self-sequential assembly, TBHP

## Abstract

We present a metal-free method for α-acetoxyarone synthesis by self-intermolecular oxidative coupling of aryl ketones using I_2_−*tert*-butyl hydroperoxide (TBHP). Under the optimum conditions, various aryl ketones gave the corresponding products in moderate to excellent yields. A series of control experiments were performed; the results suggest the involvement of radical pathways. Multiple radical intermediates were generated in situ and the overall process involved several different reactions, which proceeded self-sequentially in a single reactor. A labeling experiment using ^18^O-labeled H_2_O confirmed that the oxygen in the product was derived from TBHP, not from H_2_O in the TBHP solvent.

## Introduction

**Scheme 1:**
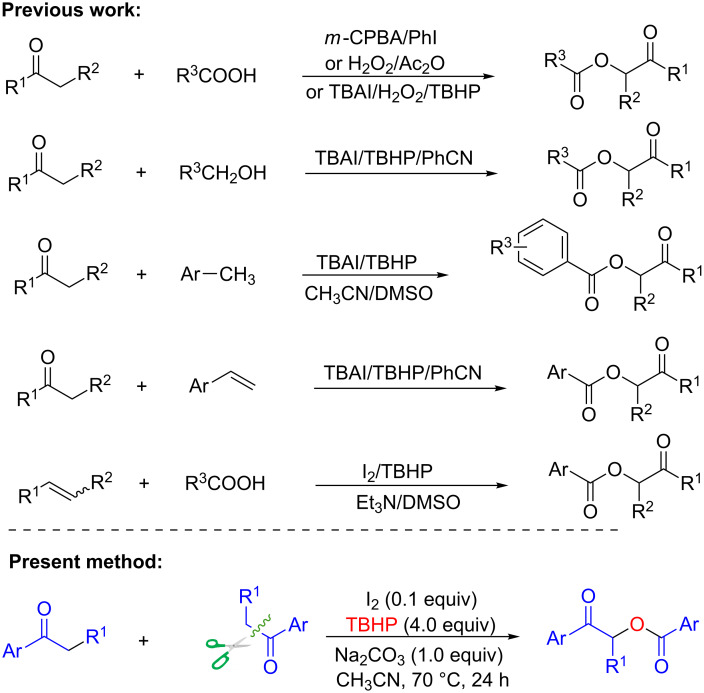
Previous and present approaches.

In recent years, α-acetoxyaryl ketones have attracted considerable interest because this structural motif is found in a variety of biologically active natural products and pharmaceuticals, and α-acetoxyaryl ketones are widely used as synthetic intermediates [1–5]. Traditional methods for the preparation of α-acyloxy ketones focus on the substitution reactions of α-halo carbonyl compounds with alkaline carboxylates or carboxylic acids [6–7], and transition-metal-catalyzed direct oxidative coupling reactions of carbonyl compounds with carboxylic acids (or their surrogates) [8–9]. Recently, robust approaches using organohypervalent iodine reagents and peroxide-mediated oxidative coupling have been developed [10–11]. Although impressive progress has been made [12], examples of the synthesis of α-acetoxyaryl ketones through self-intermolecular oxidative coupling of aryl ketones are still rare. Yan and coworkers reported the preparation of α-acyloxyaryl ketones from aryl ketones using a Pybox-copper(II) catalyst [13]. However, the substrate scope was limited to α-substituted aryl ketones, and acetophenones were unsuitable for this conversion. In addition, this method requires harsh catalytic conditions, using scarce iron and copper complexes. The development of novel metal-free methods for the preparation of α-acetoxyaryl ketones is therefore an attractive target for organic chemists. Simple, inexpensive, and metal-free methods [14–15], involving safe and clean oxidation procedures, need to be developed. Here, we report a metal-free, novel, and efficient self-intermolecular oxidative coupling procedure for the synthesis of α-acetoxyaryl ketones from aryl ketones using I_2_ and *tert*-butyl hydroperoxide (TBHP) [16–18] (Scheme 1). Several oxidative cross-coupling methods have been developed for the synthesis of α-acetoxy ketones from ketone derivatives and carboxylic acids [10], benzylic alcohols [19], toluene derivatives [20–21] and alkenes [22–23] using TBHP as the oxidant (Scheme 1). However, to the best of our knowledge, this is the first example of the use of TBHP as the oxidant for the construction of α-acetoxyaryl ketones from aryl ketones via self-intermolecular oxidative coupling.

## Results and Discussion

In our first attempt, the reaction of acetophenone (**1a**) in the presence of an I_2_–TBHP system gave the desired product **2a** in 46% yield. The yield increased to 71% when the reaction time was prolonged to 24 h (Table 1, entries 1–3). The reaction did not occur in the absence of I_2_ or Na_2_CO_3_, indicating that these species both play important roles in this reaction (Table 1, entries 4 and 5). The reaction was almost unaffected by the solvent (Table 1, entries 3, 6−8). Acetonitrile was slightly more effective than the other solvents tested. An increase in the amount of TBHP from 2.0 equiv to 4.0 equiv significantly affected the reaction efficiency, leading to a pronounced increase in the yield (Table 1, entry 9). Further increasing the TBHP loading did not have any beneficial effect (Table 1, entry 10). An increase in the amount of I_2_ from 0.1 equiv to 0.5 equiv did not affect product formation (Table 1, entry 11). However, decreasing the amount of Na_2_CO_3_ from 1.0 equiv to 0.1 equiv significantly decreased the product yield. The effects of other peroxides, i.e., di-*tert*-butyl peroxide (DTBP), benzoyl peroxide, dicumyl peroxide (DCP), cumene hydroperoxide (CHP), potassium hydrogen persulfate, and 3-chloroperoxybenzoic acid (*m*-CPBA), on the reaction were investigated. All these peroxides gave sluggish reactions with poor yields, except *m*-CPBA, which gave the desired product **2a** in 81% yield (Table 1, entries 13–18). Finally, we investigated the effect of reaction temperature to this transformation, which indicated that the optimum reaction temperature is: 70 °C (Table 1, entries 19 and 20).

**Table 1:** Optimization studies^a^.

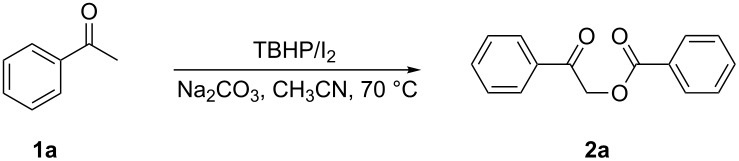				

Entry	peroxide (2.0 equiv)	solvent	Time (h)	Yield%^b^

1	TBHP	CH_3_CN	12	46
2	TBHP	CH_3_CN	24	71

3	TBHP	CH_3_CN	36	71
4^c^	TBHP	CH_3_CN	24	0
5^d^	TBHP	CH_3_CN	24	0
6	TBHP	dioxane	24	70
7	TBHP	DCE	24	68
8	TBHP	cyclohexane	24	63
9	TBHP (4.0)	CH_3_CN	24	84
10	TBHP (6.0)	CH_3_CN	24	84
11^e^	TBHP (4.0)	CH_3_CN	24	84
12^f^	TBHP (4.0)	CH_3_CN	24	33
13	DTBP (4.0)	CH_3_CN	24	23
14	benzoyl peroxide (4.0)	CH_3_CN	24	47
15	DCP (4.0)	CH_3_CN	24	29
16	CHP (4.0)	CH_3_CN	24	11
17	K_2_S_2_O_8_ (4.0)	CH_3_CN	24	11
18	*m*-CPBA (4.0)	CH_3_CN	24	81
19^g^	TBHP (4.0)	CH_3_CN	24	trace
20^h^	TBHP (4.0)	CH_3_CN	24	84

^a^Reaction conditions: **1a** (0.5 mmol), I_2_ (0.1 equiv), TBHP (2.0 equiv), Na_2_CO_3_ (1.0 equiv), solvent (2.0 mL); ^b^GC yield; ^c^without I_2_; ^d^without Na_2_CO_3_; ^e^I_2_: 0.5 equiv; ^f^Na_2_CO_3_: 0.1 equiv; ^g^reaction temperature: rt; ^h^reflux.

**Scheme 2:**
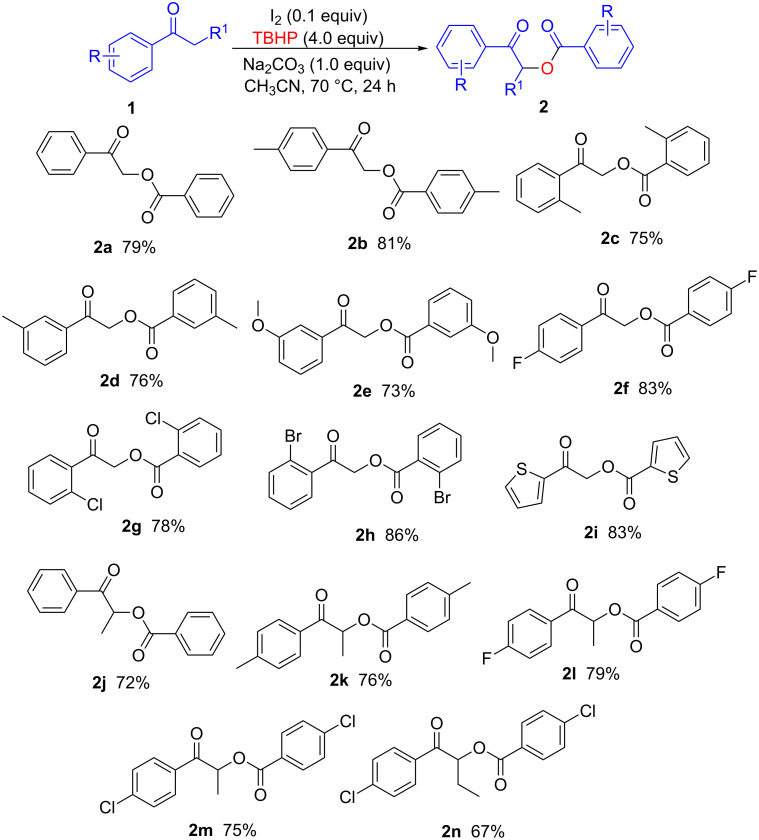
Substrate scope. (All of these reactions were carried out on a 2.0 mmol scale using CH_3_CN (2.0 mL) as a solvent.)

After the optimization study, the generality of the optimum conditions with various substituted aryl ketones was investigated (Scheme 2). Initially, acetophenone derivatives **1a**–**h** were used; various electron-donating (i.e., methyl and methoxy) and electron-withdrawing (i.e., F^−^, Br^−^, and Cl^−^) substituents were well-tolerated under our reaction conditions. Acetophenones bearing electron-withdrawing substituents performed slightly better in this reaction than those bearing electron-donating substituents, and afforded the desired product in relatively high yields (**2f**, **2g**, and **2h**).

The position of a given substituent on the phenyl ring of acetophenone affected the reaction slightly, and *para*-substituted acetophenones gave better results than *ortho-* and *meta*-substituted acetophenones (**2b**, **2c**, and **2d**). The scope of this reaction was extended by varying the aliphatic part of the arone (**1j–n**); for example, propiophenones and butyrophenones all reacted as anticipated to give the desired α-acetoxyaryl ketones **2j–n** in moderate yields. In addition, different substituents on the phenyl ring had no discernible impact on the outcome. 1-(Thiophen-2-yl)ethanone (**1i**), which has a heteroaryl functionality, gave **2i** in 83% isolated yield.

**Scheme 3:**
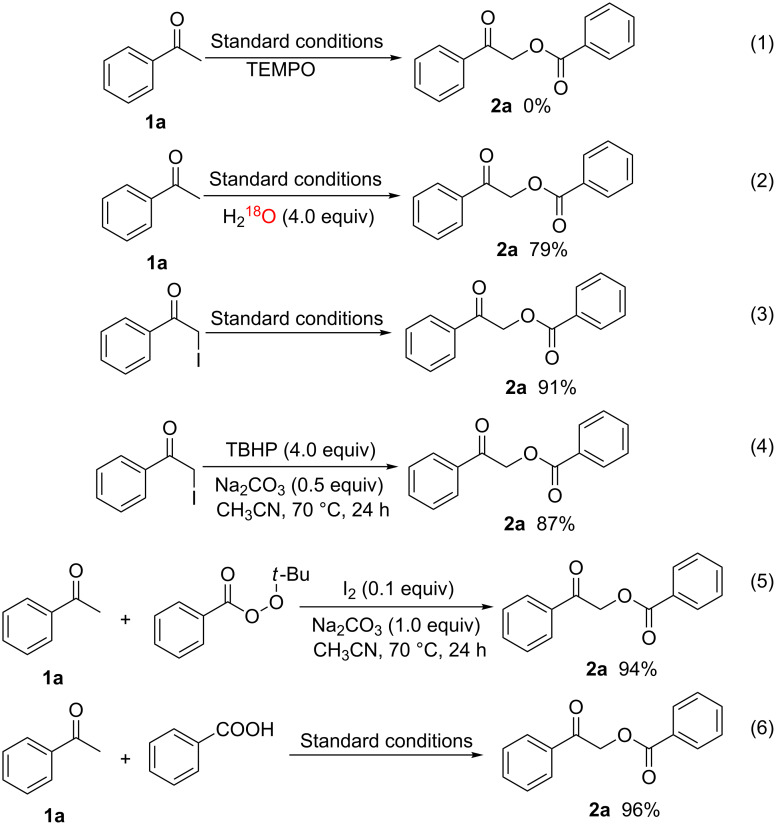
Control reactions for clarifying the mechanism.

A series of control experiments were performed to clarify the reaction mechanism (Scheme 3). When the reactions were performed in the presence of an excess of the free-radical scavenger 2,2,6,6-tetramethylpiperidine-*N*-oxyl, product formation was completely suppressed (Scheme 3, reaction 1), indicating that a radical pathway may be involved in this reaction. The oxygen source was identified by performing the reaction with excess ^18^O-labeled H_2_^18^O; **2a** was obtained in 79% yield, with no ^18^O in the product; this excludes the possibility of the oxygen being derived from H_2_O in the TBHP solvent (Scheme 3, reaction 2).When 2-iodo-1-phenylethanone was used as a surrogate of **1a** under the optimum conditions or in the absence of I_2_, **2a** was isolated in 91% and 87% yields, respectively (Scheme 3, reactions 3 and 4). We also observed that **2a** was obtained in almost quantitative yields when **1a** was reacted with *tert*-butylperoxybenzoate (TBPB) or benzoic acid under the standard conditions (Scheme 3, reactions 5 and 6). These results suggest that 2-iodo-1-phenyl ketone, TBPB, and benzoic acid are generated in situ from **1a** as intermediates.

**Scheme 4:**
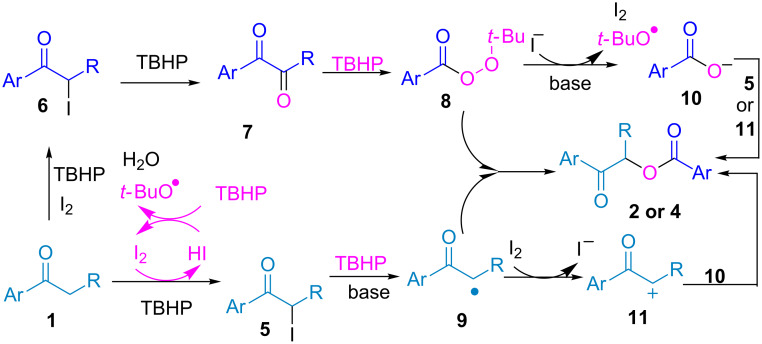
Plausible mechanism.

The mechanism has not yet been clarified in detail. A probable catalytic cycle is proposed in Scheme 4 based on the above experimental results and previous literature reports. The process begins with the formation of α-iodoaryl ketones **5** and **6** via iodination of aryl ketones with I_2_ and TBHP [24–25]. An I^−^/I_2_ redox cycle promotes *tert*-butoxyl and *tert*-butylperoxyl radical formation from TBHP [26–28]. In the presence of TBHP and I_2_, α-iodoaryl ketones **5** and **6** are oxidized to a 1,2-diketone intermediate **7** and an α-carbonyl radical **9**, which can be further transformed to *tert*-butyl perester **8** and cation **11** [22]. The I^−^ anion can be reoxidized by *tert*-butyl perester **8** to regenerate I_2_, a *tert*-butoxyl radical, and an aromatic acid anion under alkaline conditions. Finally, the reactions between intermediates **8** and **9, 10** and **11** or **5** all afford the final product, according to previous reports [22,29].

## Conclusion

In summary, we have developed an efficient, novel, and metal-free synthesis of α-acetoxyaryl ketones from aryl ketones using I_2_−TBHP. A facile α-acylation reaction involving self-intermolecular oxidative coupling of aryl ketones was observed for the first time in the presence of I_2_−TBHP. Multiple radical intermediates are generated in situ, and the overall process involves several different reactions, which proceed self-sequentially in a single reactor. The reaction conditions are mild and the substrate scope is broad. This method has good potential applications in organic synthesis and medicinal chemistry. The inside of the reaction mixture has not been studied in depth, but we have begun mechanistic studies.

## Supporting Information

File 1 Full experimental details and copies of NMR spectral data.
