# Supplementary material for: α-Acetoxyarone synthesis via iodine-catalyzed and tert-butyl hydroperoxide-mediateded self-intermolecular oxidative coupling of aryl ketones
Source: Beilstein J Org Chem. 2017 Jun 6;13:1079–84. doi: 10.3762/bjoc.13.107 (PMC5480364; doi:10.3762/bjoc.13.107)
Supplement: File 1 — Full experimental details and copies of NMR spectral data. [file Beilstein_J_Org_Chem-13-1079-s001.pdf]

Supporting Information

for

α-Acetoxyarone synthesis via iodine-catalyzed and *tert*-butyl hydroperoxide-mediateded self-intermolecular oxidative coupling of aryl ketones

Liquan Tan*, Cui Chen, Weibing Liu

Address: College of Chemical Engineering, Guangdong University of Petrochemical Technology, 2 Guandu Road, Maoming 525000, P. R. China. Fax: +86-668-2923575; Tel: +86-668-2923444

Email: Liquan Tan - touching522@gdupt.edu.cn

*Corresponding author

Full experimental details and copies of NMR spectral data

General Information. All the reactions were carried out at 70 °C for 24 h in a round-bottom flask equipped with a magnetic stir bar. Unless otherwise stated, all reagents and solvents were purchased from commercial suppliers and used without further purification. ^{1}H NMR and ^{13}C NMR spectra were recorded on a 400 MHz spectrometer in solutions of $CDCl_3$ using tetramethylsilane as the internal standard; δ values are given in ppm, and coupling constants (*J*) in Hz. All melting points are uncorrected. HRMS were obtained on a Q-TOF micro spectrometer.

Typical procedure: benzoic acid, 2-phenyl-2-oxoethyl ester (2a). A mixture of acetophenone (**1a**) (240 mg, 2.0 mmol), I_2 (50.8 mg, 0.2 mmol), TBHP (1032 mg, 8.0 mmol, 70% in water), Na_2CO_3 (212 mg, 2.0 mmol), and CH_3CN (2.0 mL) was added successively into a round-bottom flask, and the resulting solution was stirred for 24 h at 70 °C. The mixture was purified by column chromatography on silica gel to afford product **2a** with PE/ethyl acetate = 20/1 as the eluent.

Benzoic acid 2-phenyl-2-oxoethyl ester (2a) [1]

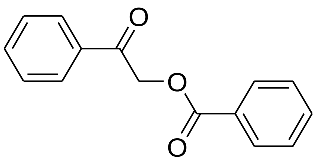

Yield: 79%; ^{1}H NMR ($CDCl_3$, 400 MHz) δ 8.15 (d, *J* = 8.4 Hz, 2H), 7.97 (d, *J* = 8.0 Hz, 2H), 7.61 (m, 2H), 7.50 (m, 4H), 5.59 (s, 2H); ^{13}C NMR ($CDCl_3$, 100 MHz) δ 192.1, 166.0, 134.2, 133.9, 133.4, 130.0, 129.4, 128.9, 128.4, 127.8, 66.5.

4-Methylbenzoic acid 2-(4-methylphenyl)-2-oxoethyl ester (2b) [2]

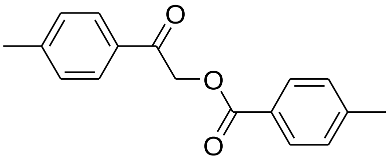

Yield: 81%; ^{1}H NMR ($CDCl_3$, 400 MHz) δ 8.03 (d, *J* = 8.4 Hz, 2H), 7.87 (d, *J* = 8.0 Hz, 2H), 7.30 (d, *J* = 8.0 Hz, 2H), 7.26 (d, *J* = 8.0 Hz, 2H), 5.54 (s, 2H), 2.438 (s, 3H), 2.434 (s, 3H); ^{13}C NMR ($CDCl_3$, 100 MHz) δ 191.8, 166.1, 144.8, 144.0, 131.8, 130.0, 129.5, 129.1, 127.9, 127.6, 66.3, 21.79, 21.74.

2-Methylbenzoic acid 2-(2-methylphenyl)-2-oxoethyl ester (2c)

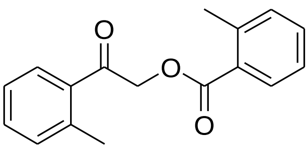

Yield: 75%; Pale yellow oil; ^{1}H NMR ($CDCl_3$, 400 MHz) δ 8.05 (d, *J* = 8.0 Hz, 1H), 7.71 (d, *J* = 8.0 Hz, 1H), 7.44 (m, 2H), 7.30 (m, 4H), 5.40 (s, 2H), 2.63 (s, 3H), 2.56 (s, 3H); ^{13}C NMR ($CDCl_3$, 100 MHz) δ 195.9, 166.9, 140.6, 139.0, 132.3, 132.2, 132.1, 131.8, 131.6, 130.9, 128.8, 128.1, 125.9, 125.7, 67.5, 21.6, 21.1; HRMS (ESI): calcd for $C_{17}H_{16}NaO_3$: [M+Na^{+}] 291.0992, found 291.0989.

3-Methylbenzoic acid 2-(3-methylphenyl)-2-oxoethyl ester (2d)

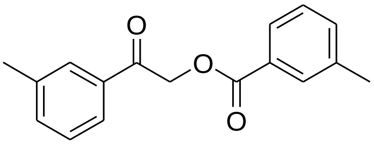

Yield: 76%; Pale yellow oil; ^{1}H NMR ($CDCl_3$, 400 MHz) δ 7.97 (m, 2H), 7.79 (m, 2H), 7.39 (m, 4H), 5.56 (s, 2H), 2.44 (s, 3H), 2.42 (s, 3H); ^{13}C NMR ($CDCl_3$, 100 MHz) δ 192.3, 166.2, 138.7, 138.2, 134.6, 134.3, 134.1, 130.5, 129.3, 128.7, 128.3, 127.1, 125.0, 66.4, 21.3, 21.2; HRMS (ESI): calcd for $C_{17}H_{16}NaO_3$: [M+Na^{+}] 291.0992, found 291.0998.

3-Methoxybenzoic acid 2-(3-methoxyphenyl)-2-oxoethyl ester (2e)

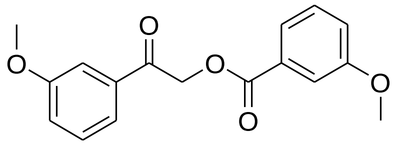

Yield: 73%; Pale yellow oil; ^{1}H NMR ($CDCl_3$, 400 MHz) δ 7.76 (s, 1H), 7.65 (s, 1H), 7.54 (m, 2H), 7.40 (m, 2H), 7.16 (m, 2H), 5.56 (s, 2H), 3.87 (s, 6H); ^{13}C NMR ($CDCl_3$, 100 MHz) δ 191.9, 165.9, 160.0, 159.5, 135.5, 130.6, 129.9, 129.4, 122.4, 120.4, 120.2, 120.1, 114.1, 112.1, 66.6, 55.5, 55.4; HRMS (ESI): calcd for $C_{17}H_{16}NaO_5$: [M+Na$^+$] 323.0890, found 323.0881.

4-Fluorobenzoic acid 2-(4-fluorophenyl)-2-oxoethyl ester (2f)

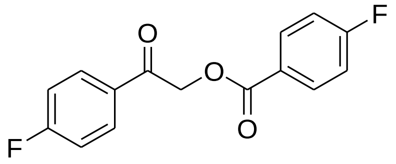

Yield: 83%; Orange oil; ^{1}H NMR ($CDCl_3$, 400 MHz) δ 8.15 (m, 2H), 8.01 (m, 2H), 7.17 (m, 4H), 5.54 (s, 2H); ^{13}C NMR ($CDCl_3$, 100 MHz) δ 190.5, 167.4 (d, $^1J_{C-F}$ = 180.0 Hz), 165.0, 164.9 (d, $^1J_{C-F}$ = 180.0 Hz), 132.5(d, $^3J_{C-F}$ = 37.6 Hz), 130.7 (d, $^4J_{C-F}$ = 12.0 Hz), 130.5 (d, $^3J_{C-F}$ = 37.6 Hz), 125.4 (d, $^4J_{C-F}$ = 12.0 Hz), 132.5(d, $^2J_{C-F}$ = 87.6 Hz), 130.7 (d, $^2J_{C-F}$ = 87.6 Hz), 66.3; HRMS (ESI): calcd for $C_{15}H_{11}F_2O_3$: [M+H$^+$] 277.0671, found 277.0690.

2-Chlorobenzoic acid 2-(2-chlorophenyl)-2-oxoethyl ester (2g) [2]

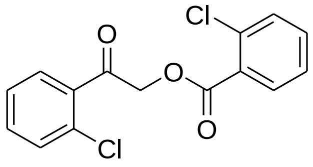

Yield: 78%; ^{1}H NMR ($CDCl_3$, 400 MHz) δ 7.98 (dd, *J* = 7.6 Hz, *J* = 2 Hz, 1H), 7.70 (dd, *J* = 7.6 Hz, *J* = 2 Hz, 1H), 7.46 (m, 4H), 7.36 (m, 2H), 5.46 (s, 2H); ^{13}C NMR ($CDCl_3$, 100 MHz) δ 195.0, 164.7, 135.8, 134.2, 133.0, 132.9, 131.9, 131.6, 131.1, 130.6, 130.2, 128.9, 127.2, 126.6, 68.9.

2-Bromobenzoic acid 2-(2-bromophenyl)-2-oxoethyl ester (2h)

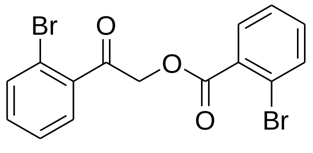

Yield: 86%; Pale yellow crystals; mp: 113-115 °C; ^{1}H NMR ($CDCl_3$, 400 MHz) δ 7.94 (dd, *J* = 7.6 Hz, *J* = 2 Hz, 1H), 7.68 (dd, *J* = 7.6 Hz, *J* = 2 Hz, 1H), 7.64 (dd, *J* = 7.6 Hz, *J* = 2 Hz, 1H), 7.57 (dd, *J* = 7.6 Hz, *J* = 2 Hz, 1H), 7.37 (m, 4H), 5.41 (s, 2H); ^{13}C NMR ($CDCl_3$, 100 MHz) δ 195.0, 164.7, 138.2,

134.4, 133.8, 133.1, 132.6, 131.9, 130.8, 129.6, 127.6, 127.2, 122.1, 119.2, 68.3; HRMS (ESI): calcd for $C_{15}H_{10}Br_2NaO_3$: [M+Na$^+$] 418.8889, found 418.8881.

2-Oxo-2-(thiophen-2-yl)ethyl thiophene-2-carboxylate (2i)

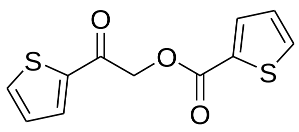

Yield: 83%; Pale yellow oil; ^{1}H NMR ($CDCl_3$, 400 MHz) δ 7.91 (dd, *J* = 4.8 Hz, *J* = 1.6 Hz, 1H), 7.81 (dd, *J* = 4.8 Hz, *J* = 1.6 Hz, 1H), 7.71 (dd, *J* = 4.8 Hz, *J* = 1.6 Hz, 1H), 7.61 (dd, *J* = 4.8 Hz, *J* = 1.6 Hz, 1H), 7.17 (dd, *J* = 4.8 Hz, *J* = 3.6 Hz, 1H), 7.13 (dd, *J* = 4.8 Hz, *J* = 3.6 Hz, 1H), 5.40 (s, 2H); ^{13}C NMR ($CDCl_3$, 100 MHz) δ 185.2, 161.4, 140.3, 134.48, 134.46, 133.2, 132.4, 132.1, 128.3, 127.9, 66.2; HRMS (ESI): calcd for $C_{11}H_8NaO_3S_2$: [M+Na$^+$] 274.9807, found 274.9799.

2-(Benzoyloxy)-1-phenyl-1-propanone (2j) [3]

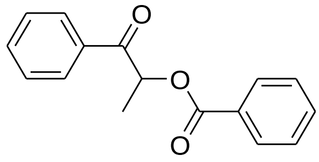

Yield: 72%; ^{1}H NMR ($CDCl_3$, 400 MHz) δ 8.10 (dd, *J* = 8.4 Hz, *J* = 1.6 Hz, 2H), 8.01 (dd, *J* = 8.4 Hz, *J* = 1.6 Hz, 2H), 7.60 (m, 2H), 7.47 (m, 4H), 6.22 (q, *J* = 7.2 Hz, 1H), 1.68 (d, *J* = 7.2 Hz, 3H); ^{13}C NMR ($CDCl_3$, 100 MHz) δ 196.7, 166.1, 134.4, 133.6, 133.3, 129.8, 129.5, 128.8, 128.5, 128.4, 71.8, 17.2.

1-Oxo-1-*p*-tolylpropan-2-yl 4-methylbenzoate (2k) [4]

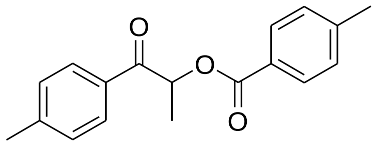

Yield: 76%; ^{1}H NMR ($CDCl_3$, 400 MHz) δ 8.00 (d, *J* = 8.0 Hz, 2H), 7.92 (d, *J* = 8.0 Hz, 2H), 7.30 (d, *J* = 8.0 Hz, 2H), 7.26 (d, *J* = 8.0 Hz, 2H), 6.19 (q, *J* = 7.2 Hz, 1H), 2.43 (s, 3H), 1.67 (d, *J* = 7.2 Hz, 3H); ^{13}C NMR ($CDCl_3$, 100 MHz) δ 196.4, 166.0, 144.4, 143.9, 131.9, 129.9, 129.4, 129.1, 128.6, 126.8, 71.6, 21.7, 17.2.

1-(4-Fluorophenyl)-1-oxopropan-2-yl 4-fluorobenzoate (2l)

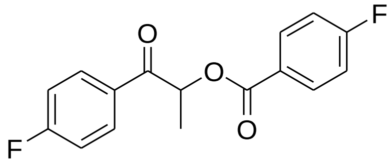

Yield: 79%; Pale yellow crystals; mp: 124-126 °C; ^{1}H NMR ($CDCl_3$, 400 MHz) δ 8.10 (dd, J = 8.4 Hz, J = 5.2 Hz, 2H), 8.03 (dd, J = 8.4 Hz, J = 5.2 Hz, 2H), 7.15 (m, 4H), 6.14 (q, J = 7.2 Hz, 1H), 1.67 (d, J = 7.2 Hz, 3H); ^{13}C NMR ($CDCl_3$, 100 MHz) δ 195.1, 167.3 (d, $^1J_{C\text{-}F}$ = 180.0 Hz), 165.0, 164.7 (d, $^1J_{C\text{-}F}$ = 180.0 Hz), 131.2 (d, $^3J_{C\text{-}F}$ = 37.6 Hz), 130.7 (d, $^4J_{C\text{-}F}$ = 12.0 Hz), 125.5 (d, $^3J_{C\text{-}F}$ = 37.6 Hz), 115.9 (d, $^4J_{C\text{-}F}$ = 12.0 Hz), 115.5 (d, $^2J_{C\text{-}F}$ = 87.6 Hz), 71.8, 17.1; HRMS (ESI): calcd for $C_{16}H_{12}F_2NaO_3$: [M+Na$^+$] 313.0647, found 313.0648.

1-(4-Chlorophenyl)-1-oxopropan-2-yl 4-chlorobenzoate (2m) [4]

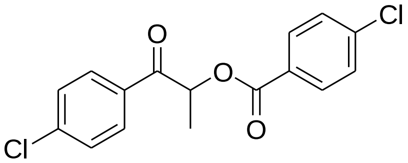

Yield: 75%; ^{1}H NMR ($CDCl_3$, 400 MHz) δ 8.02 (d, J = 8.4 Hz, 2H), 7.94 (d, J = 8.4 Hz, 2H), 7.47 (d, J = 8.4 Hz, 2H), 7.43 (d, J = 8.4 Hz, 2H), 6.13 (q, J = 7.2 Hz, 1H), 1.66 (d, J = 7.2 Hz, 3H); ^{13}C NMR ($CDCl_3$, 100 MHz) δ 195.4, 164.7, 140.2, 139.9, 132.6, 131.2, 129.9, 129.2, 128.8, 127.7, 71.9, 17.1.

1-(4-Chlorophenyl)-1-oxobutan-2-yl 4-chlorobenzoate (2n)

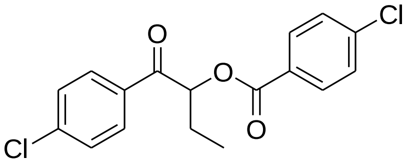

Yield: 67%; Pale yellow crystals; mp: 117-119 °C; ^{1}H NMR ($CDCl_3$, 400 MHz) δ 8.03 (d, J = 8.0 Hz, 2H), 7.94 (d, J = 8.0 Hz, 2H), 7.48 (d, J = 8.0 Hz, 2H), 7.44 (d, J = 8.0 Hz, 2H), 5.97 (t, J = 7.2 Hz, 1H), 2.02 (m, 2H), 1.11 (t, J = 7.2 Hz, 3H); ^{13}C NMR ($CDCl_3$, 100 MHz) δ 194.7, 165.3, 140.1, 139.9, 131.2, 130.5, 129.8, 129.7, 129.2, 128.8, 76.8, 29.7, 9.94; HRMS (ESI): calcd for $C_{17}H_{14}Cl_2NaO_3$: [M+Na$^+$] 359.0212, found 359.0219.

NMR spectra

Benzoic acid 2-phenyl-2-oxoethyl ester (2a)

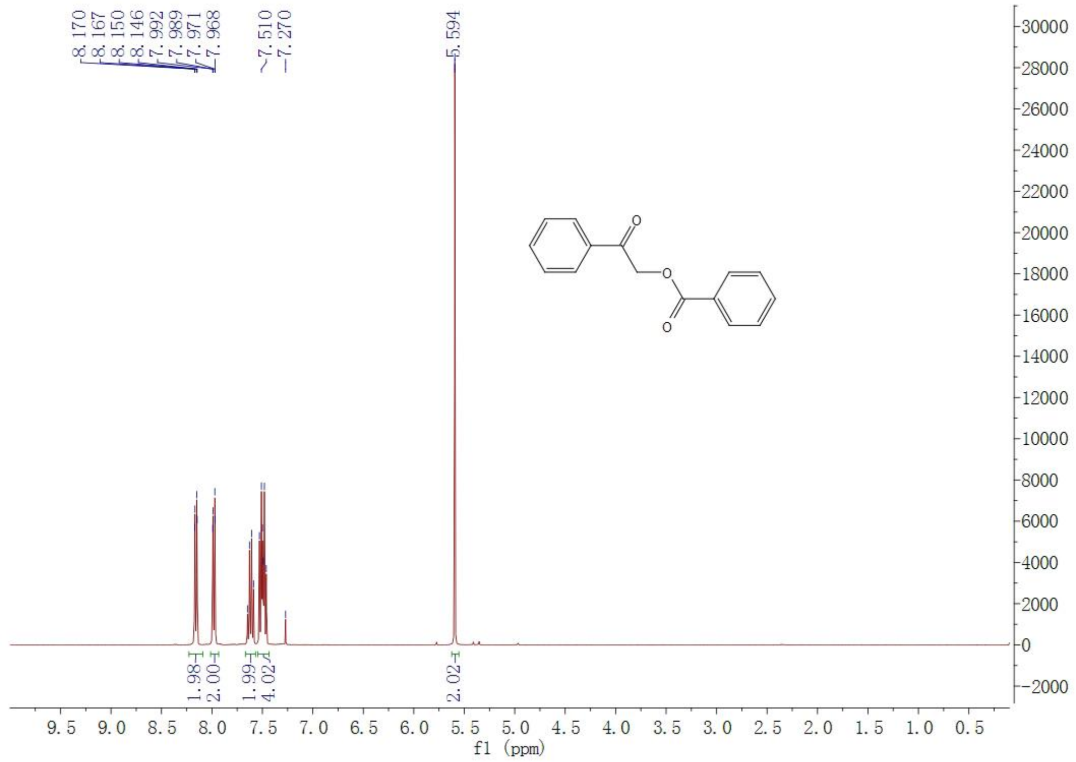

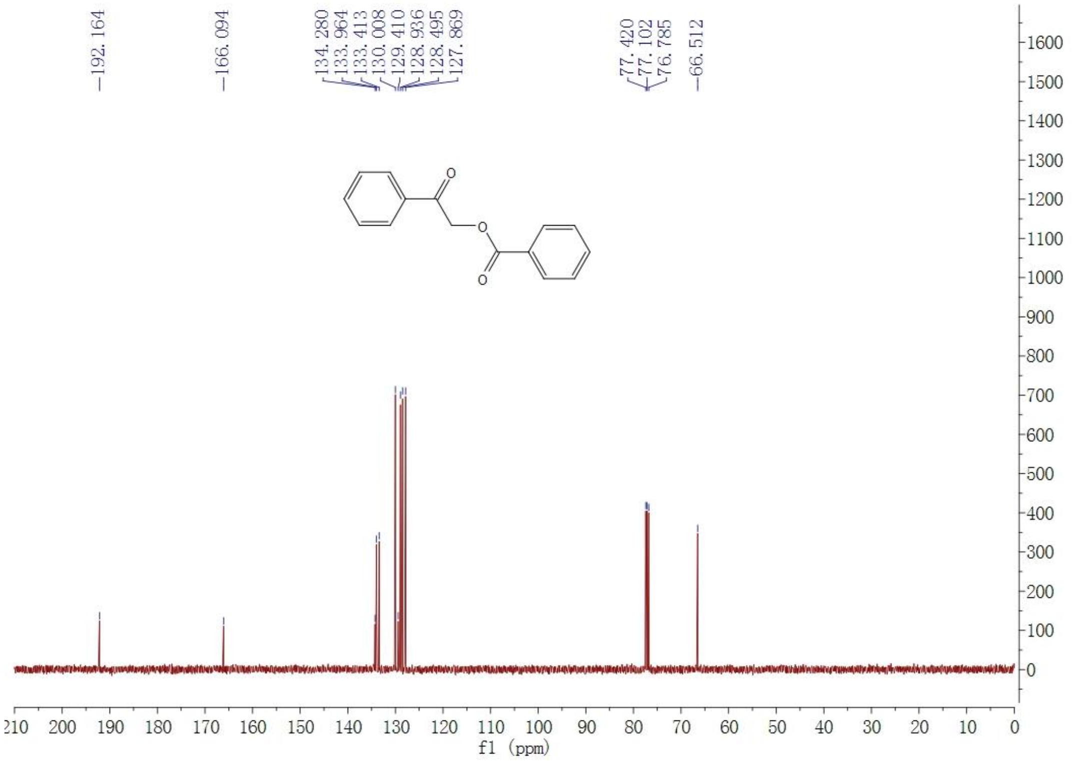

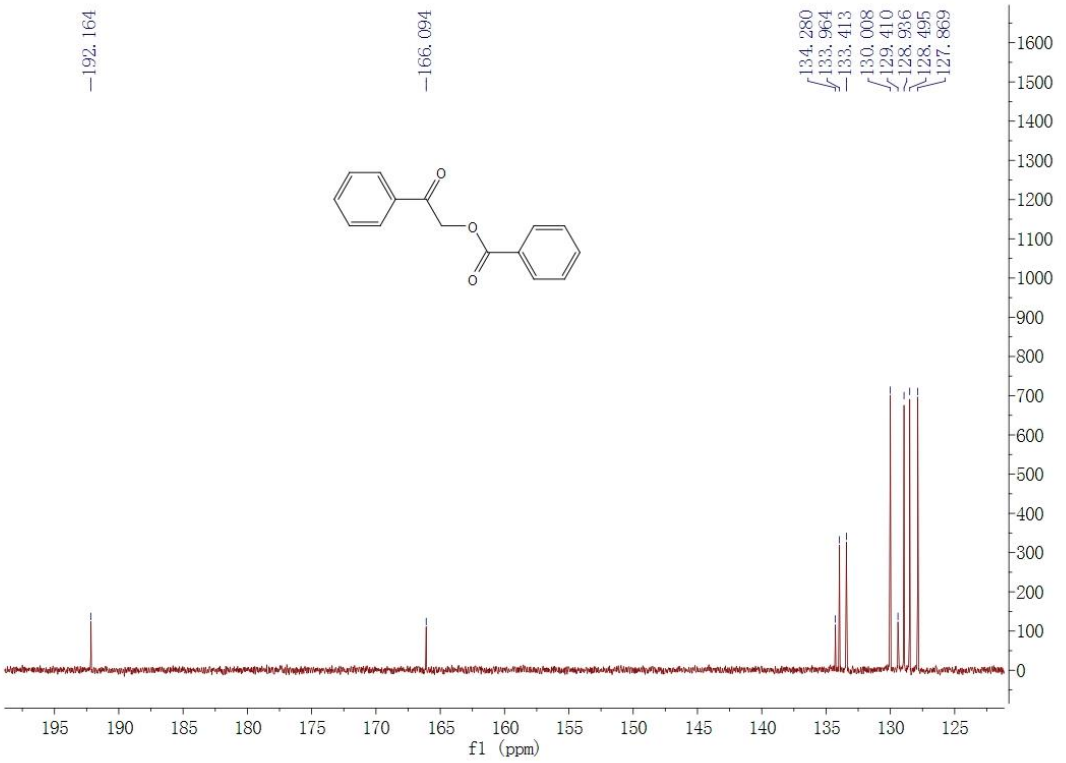

4-Methylbenzoic acid 2-(4-methylphenyl)-2-oxoethyl ester (2b)

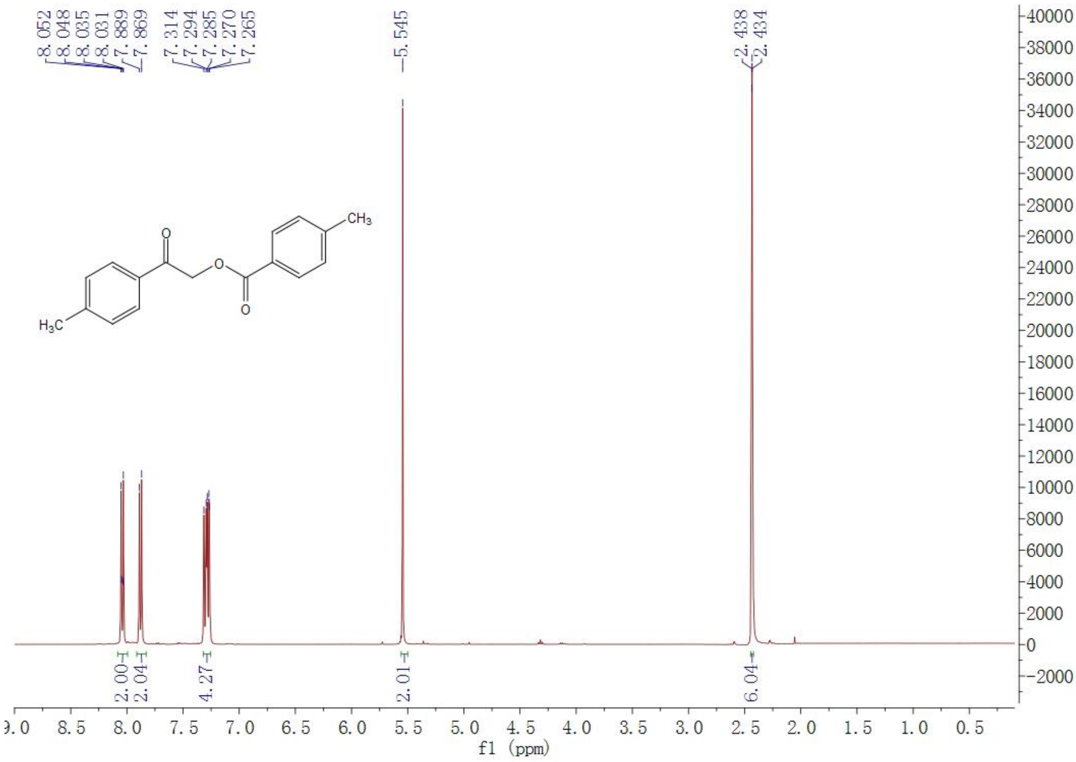

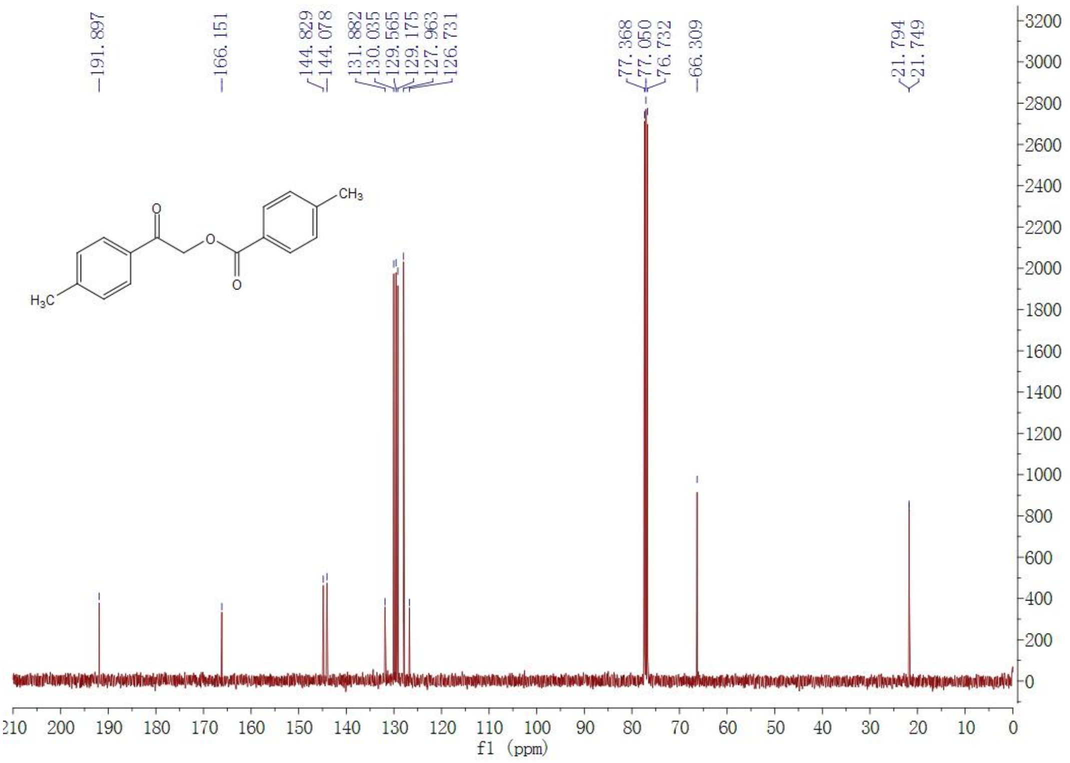
191.897
166.151
144.829
144.078
131.882
130.035
129.565
129.175
127.963
126.731
77.368
77.050
76.732
66.309
21.794
21.749
O
O
O
H_3C
CH_3
f1 (ppm)

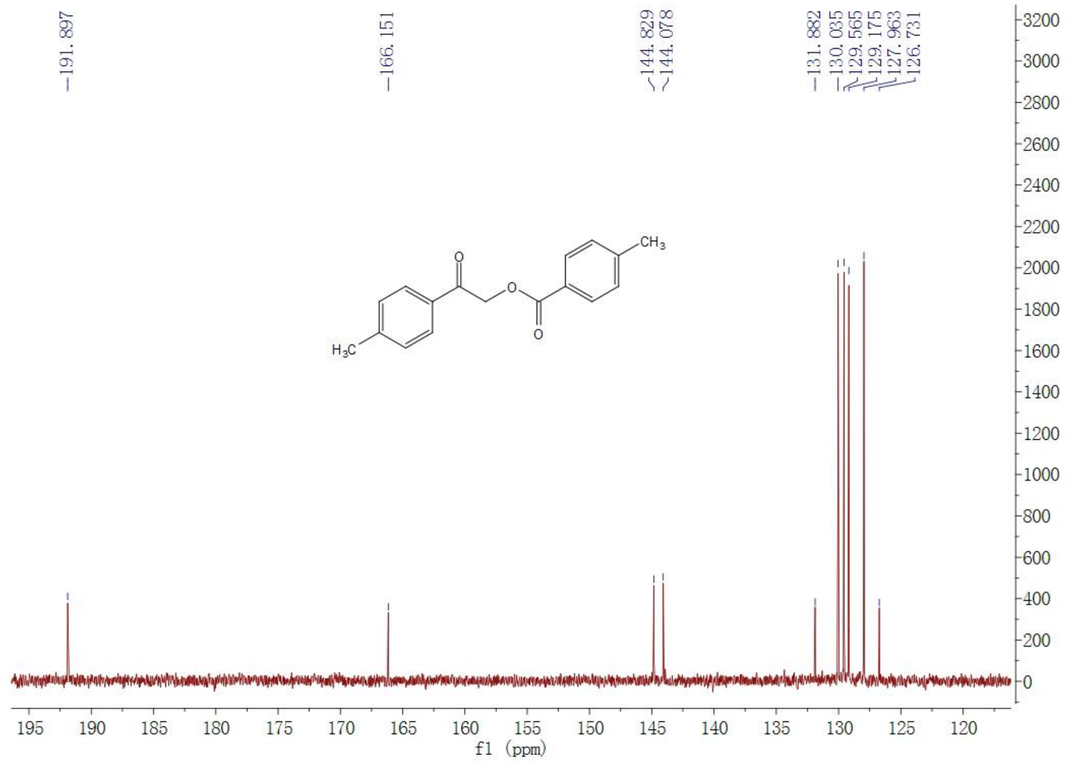
191.897
166.151
144.829
144.078
131.882
130.035
129.565
129.175
127.963
126.731
O
O
O
H_3C
CH_3
f1 (ppm)

2-Methylbenzoic acid 2-(2-methylphenyl)-2-oxoethyl ester (2c)

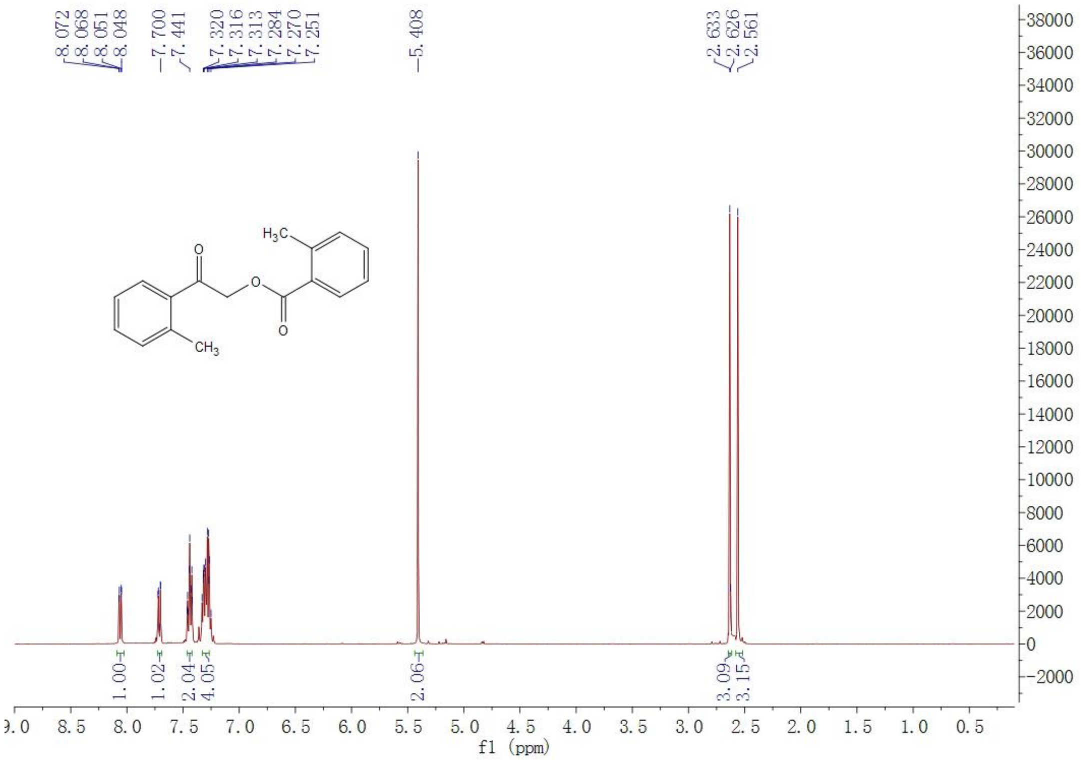

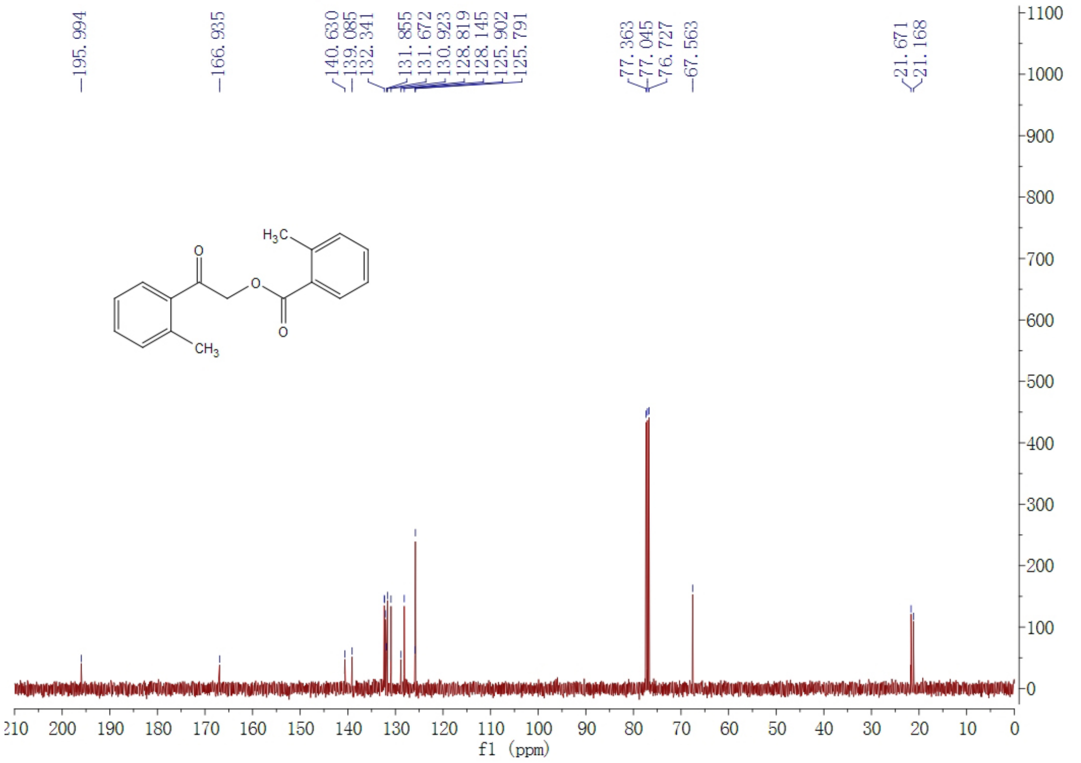

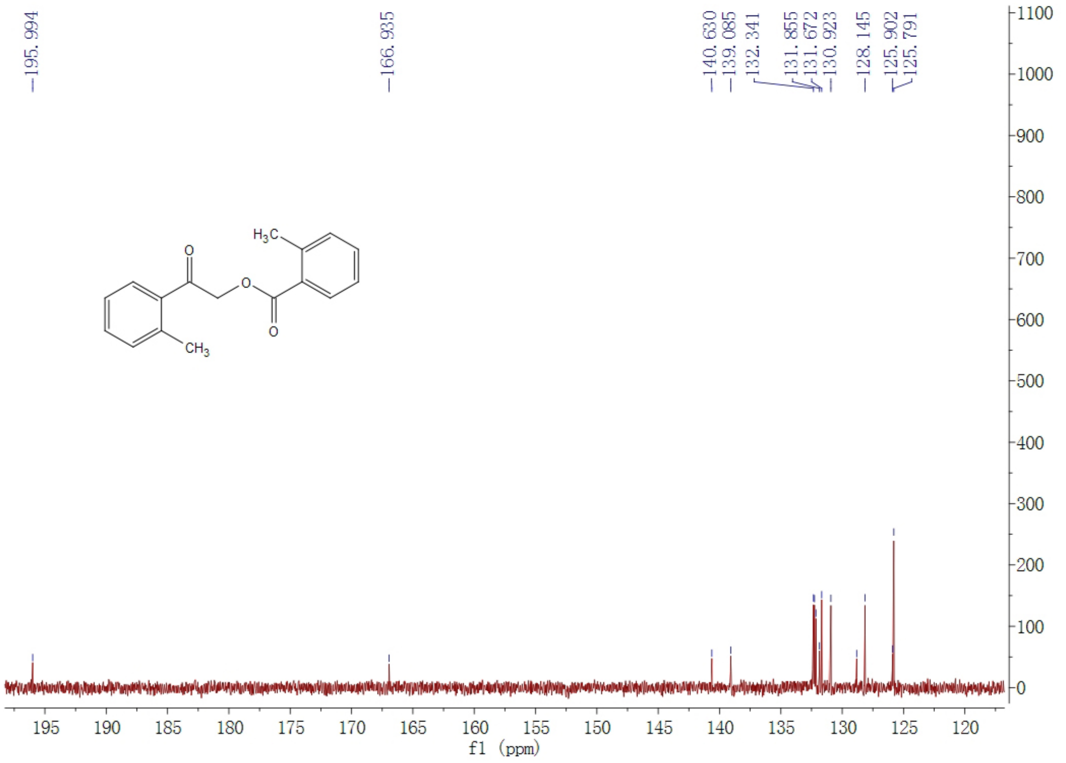

3-Methylbenzoic acid 2-(3-methylphenyl)-2-oxoethyl ester (2d)

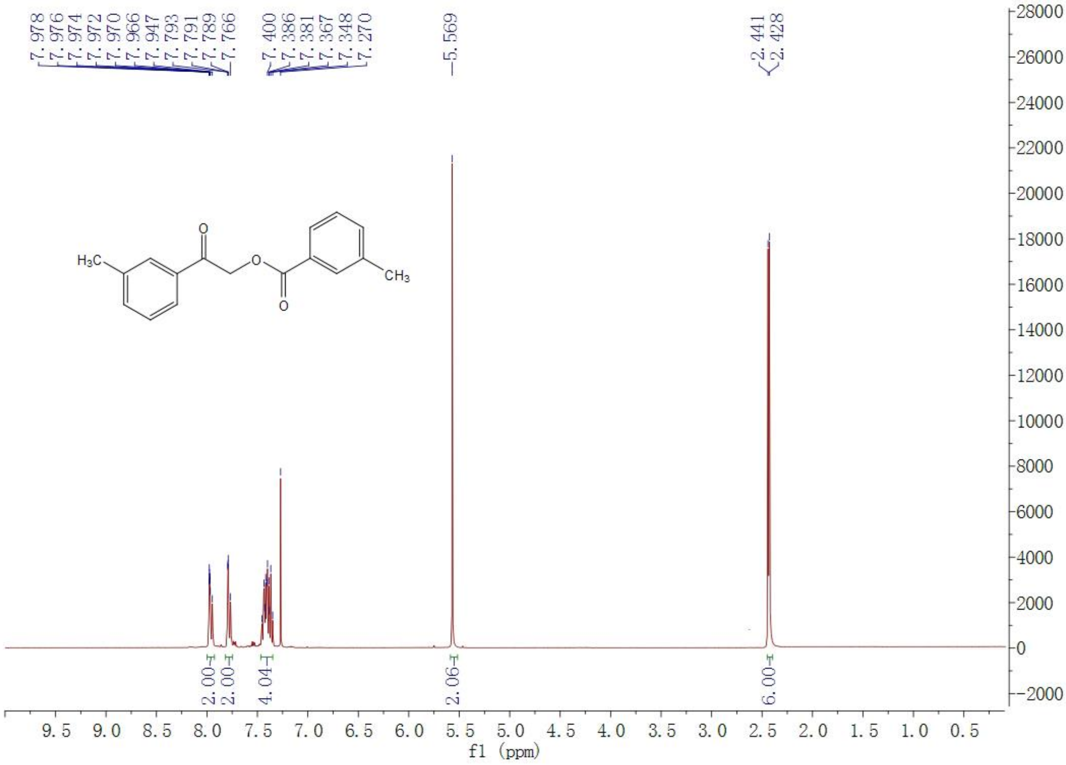

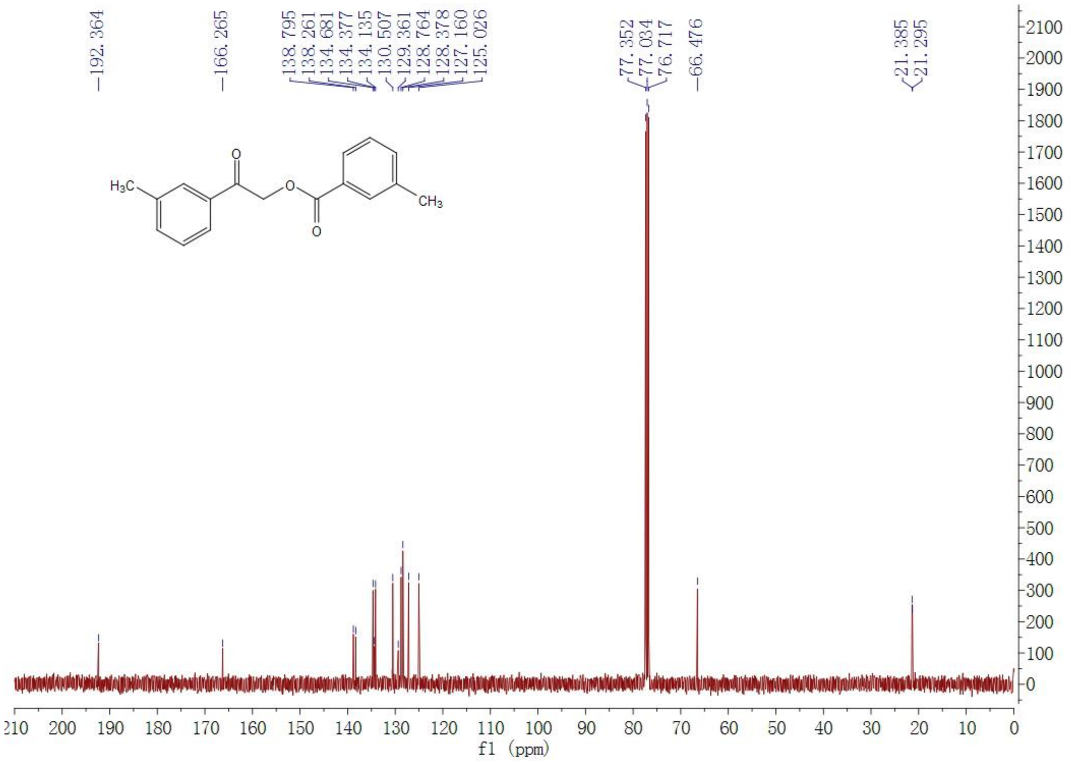
192.364
166.265
138.795
138.261
134.681
134.377
134.135
130.507
129.361
128.764
128.378
127.160
125.026
77.352
77.034
76.717
66.476
21.385
21.295
H3C
O
O
O
CH3
f1 (ppm)

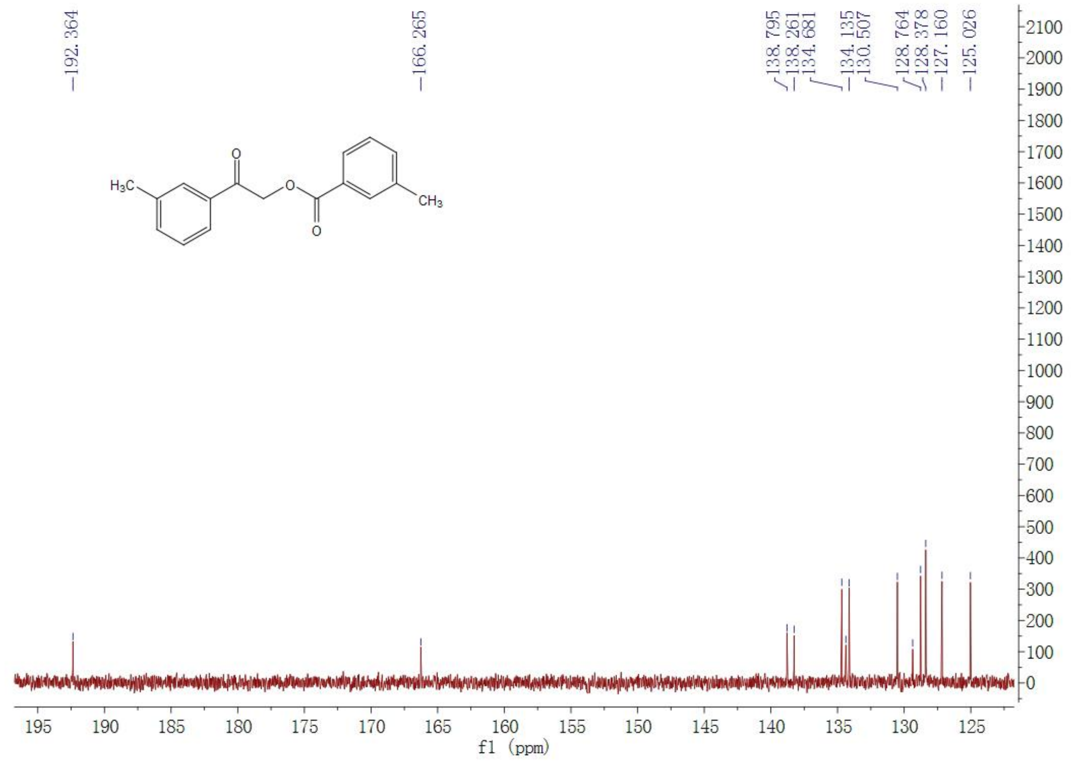
192.364
166.265
138.795
138.261
134.681
134.135
130.507
128.764
128.378
127.160
125.026
H3C
O
O
O
CH3
f1 (ppm)

3-Methoxybenzoic acid 2-(3-methoxyphenyl)-2-oxoethyl ester (2e)

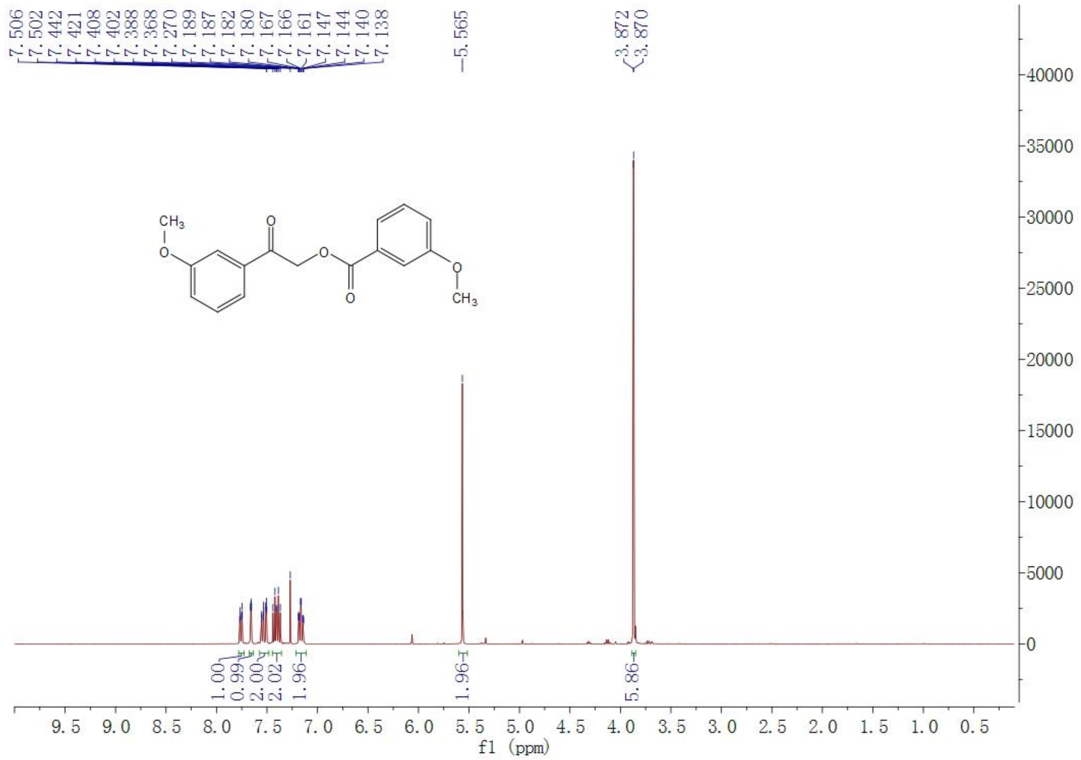

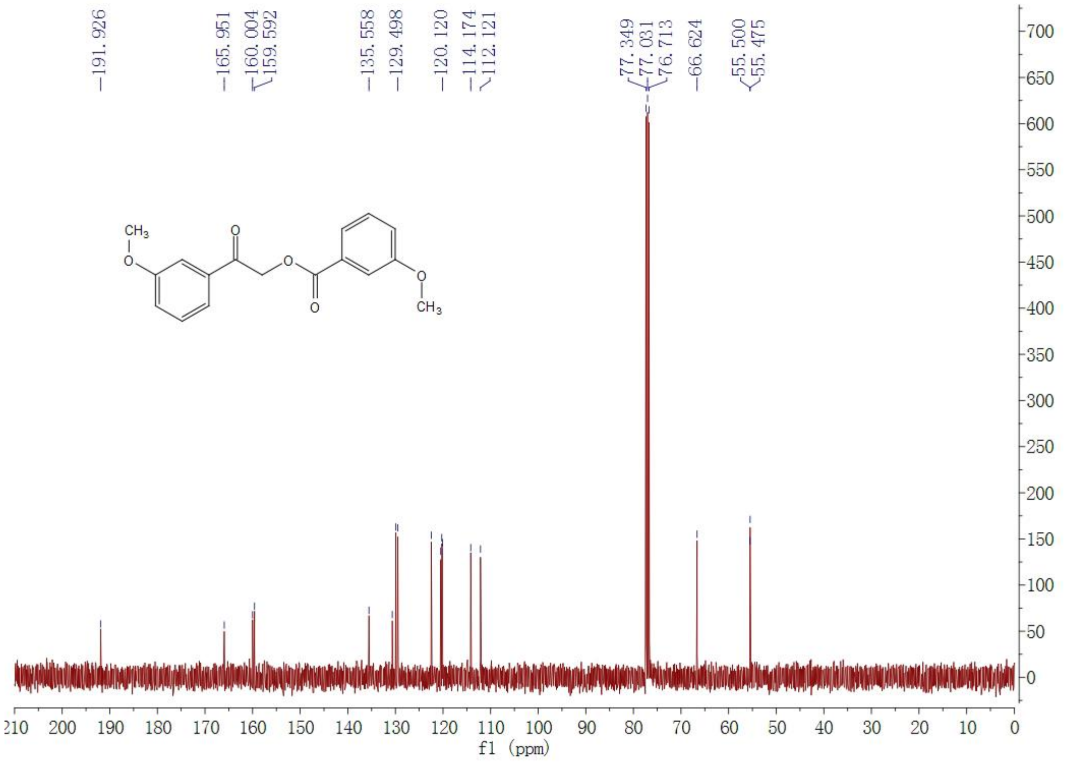

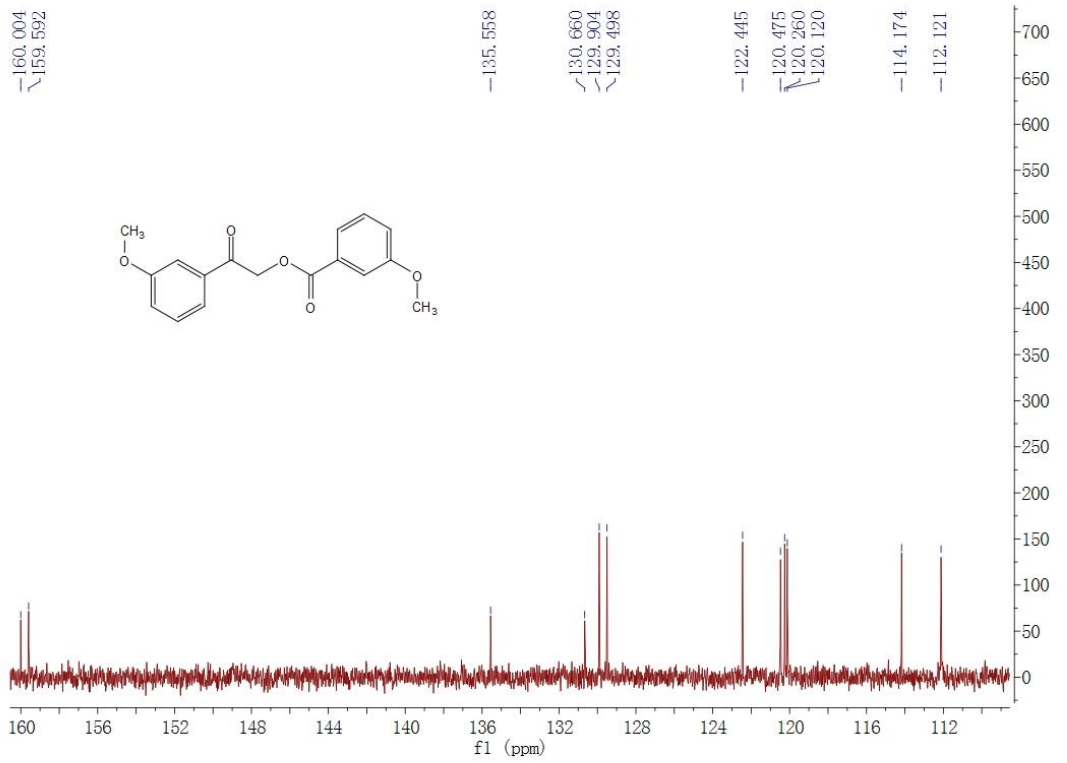

4-Fluorobenzoic acid 2-(4-fluorophenyl)-2-oxoethyl ester (2f)

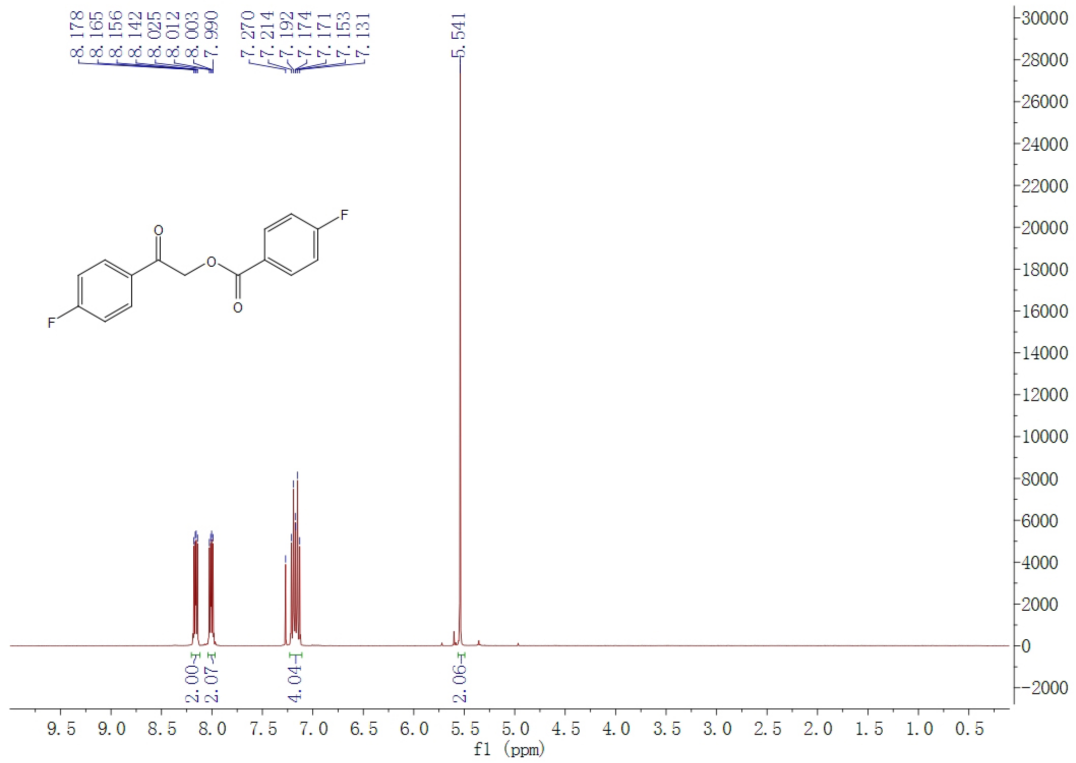

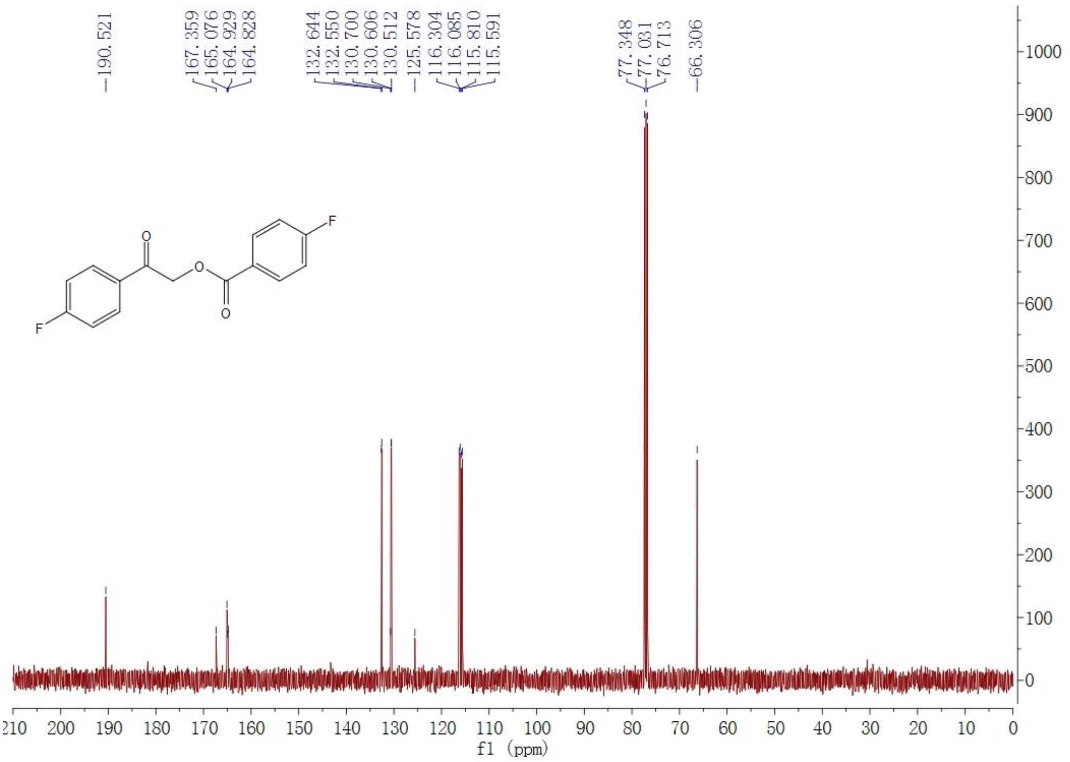

190.521
167.359
165.076
164.929
164.828
132.644
132.550
130.700
130.606
130.512
125.578
116.304
116.085
115.810
115.591
77.348
77.031
76.713
66.306
f1 (ppm)

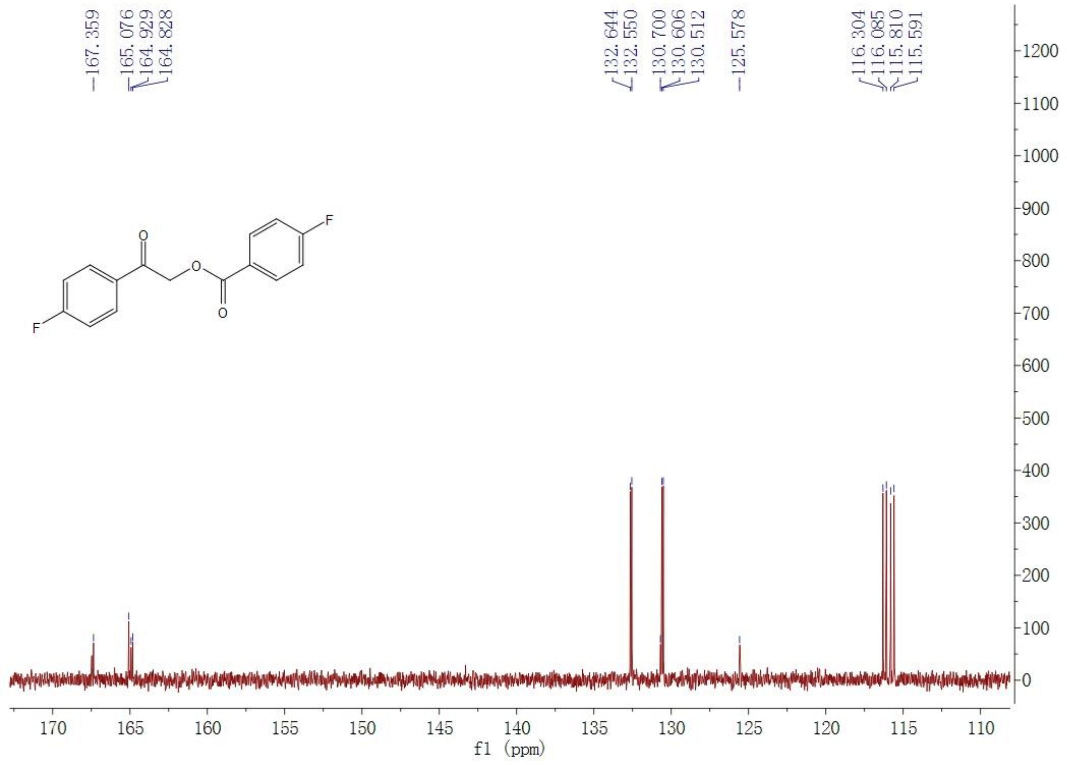

167.359
165.076
164.929
164.828
132.644
132.550
130.700
130.606
130.512
125.578
116.304
116.085
115.810
115.591
f1 (ppm)

2-Chlorobenzoic acid 2-(2-chlorophenyl)-2-oxoethyl ester (2g)

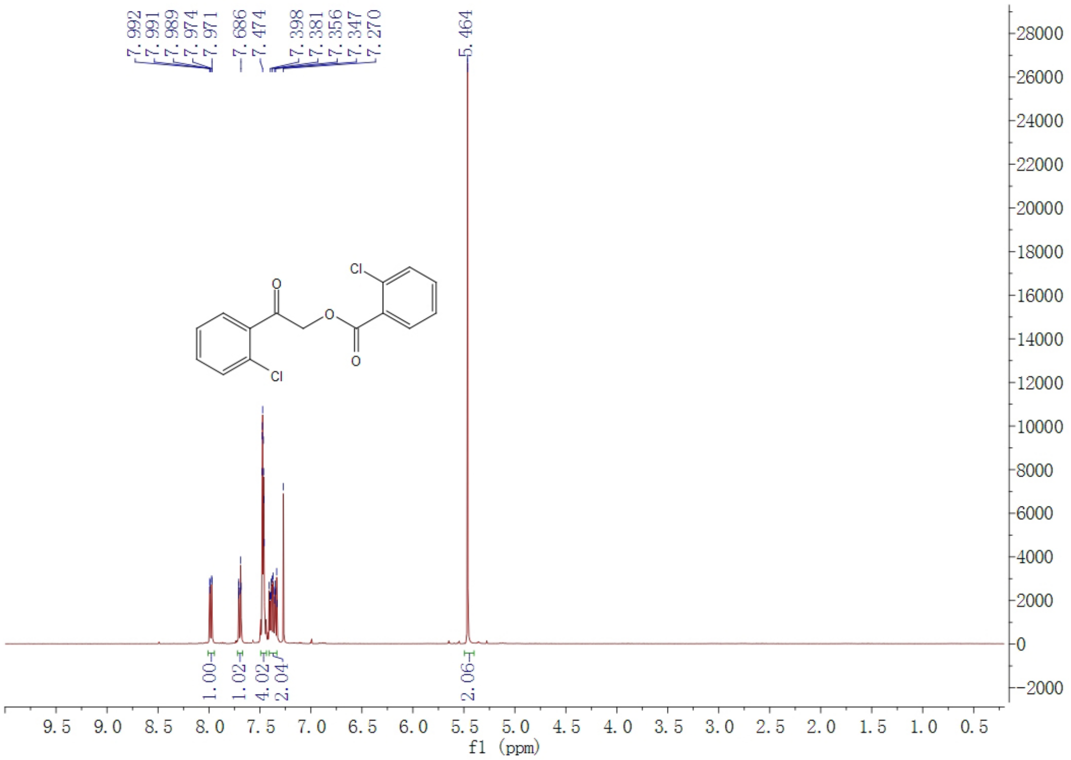

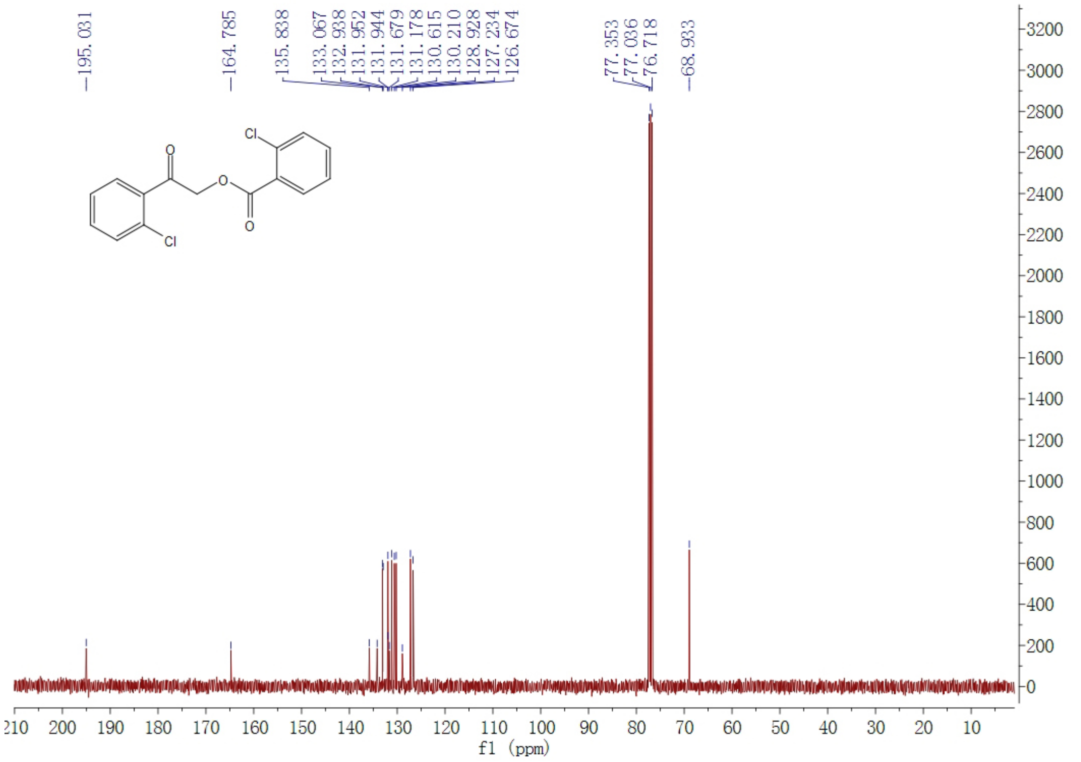

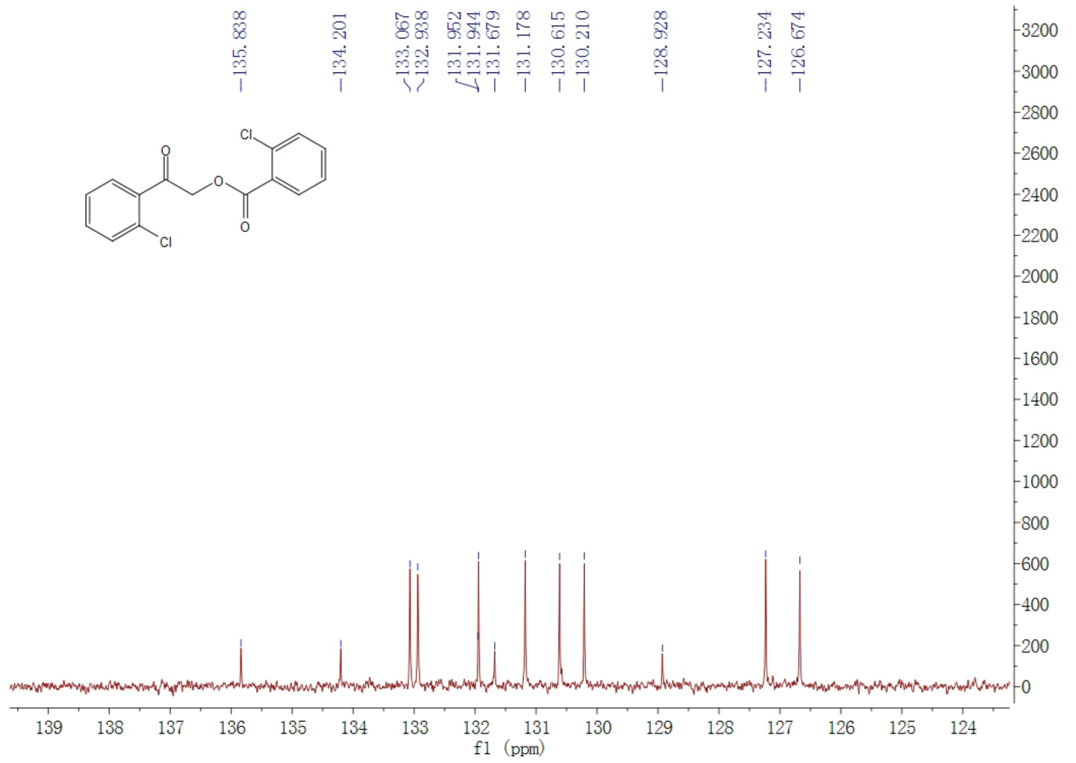

2-Bromobenzoic acid 2-(2-bromophenyl)-2-oxoethyl ester (2h)

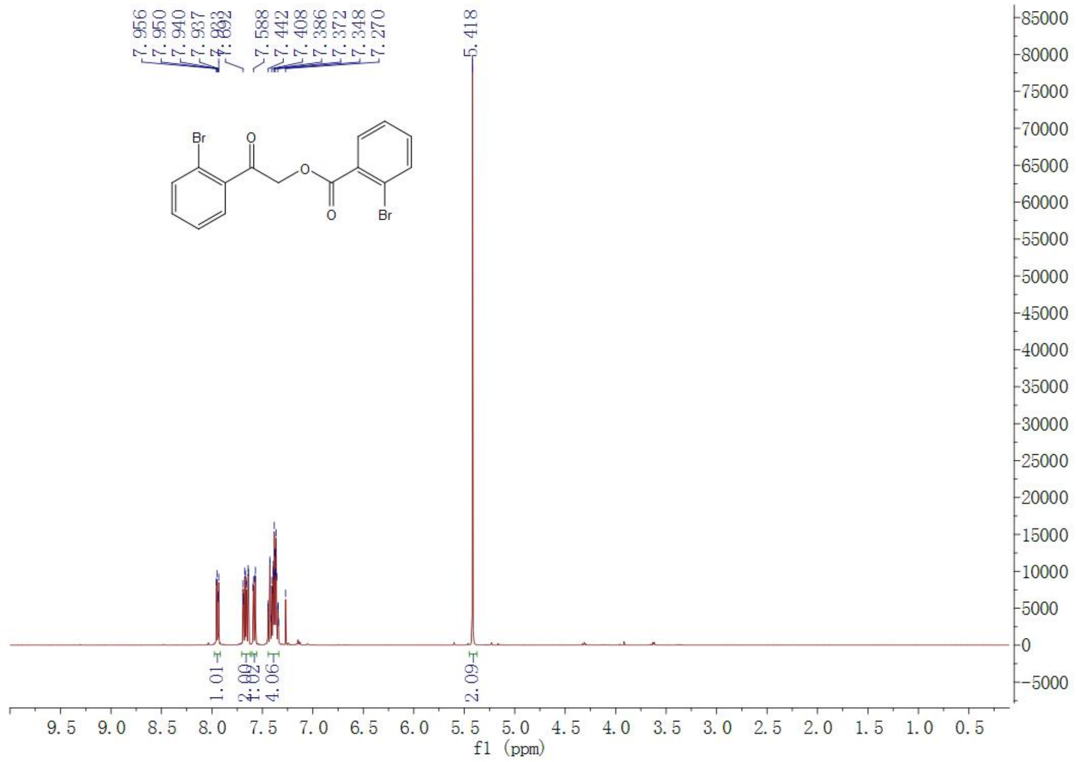

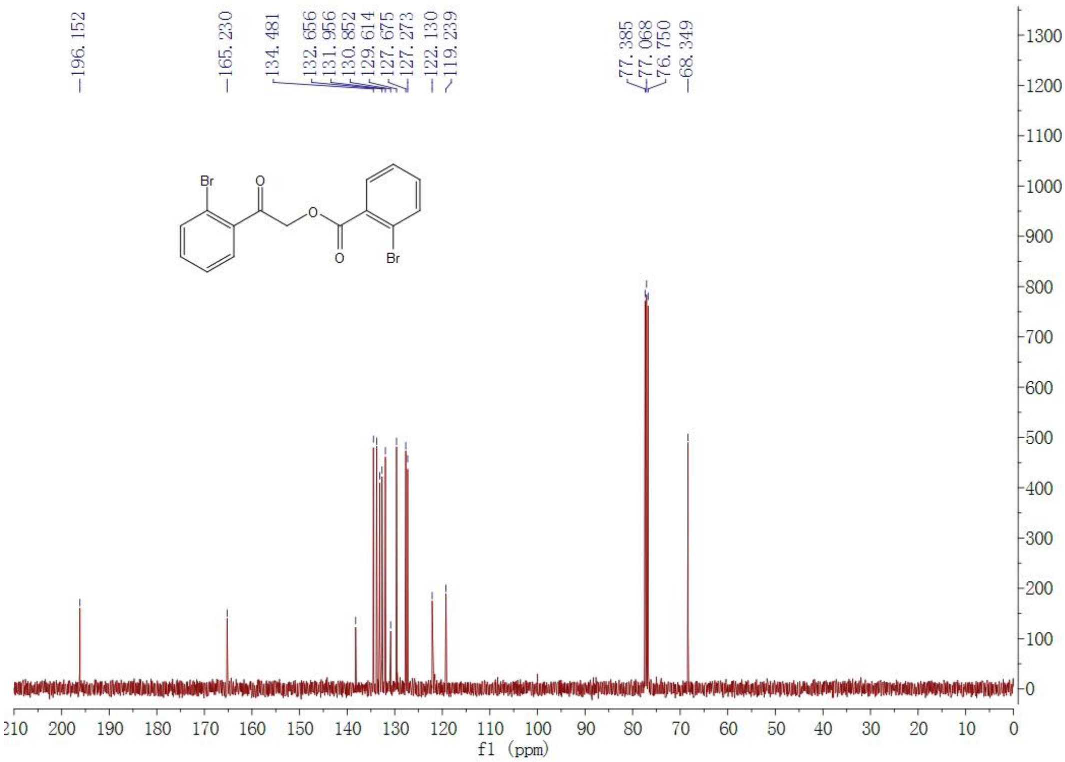

196.152
165.230
134.481
132.656
131.956
130.852
129.614
127.675
127.273
122.130
119.239
77.385
77.068
76.750
68.349
Br
O
O
O
Br
f1 (ppm)

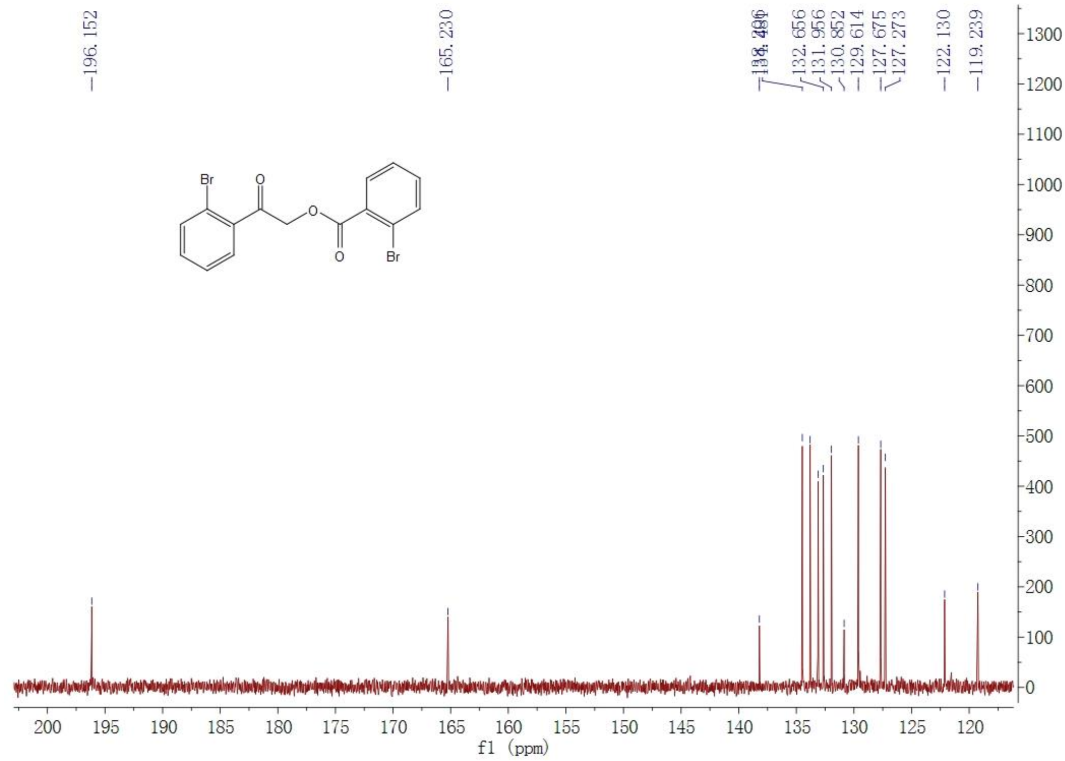

196.152
165.230
132.656
131.956
130.852
129.614
127.675
127.273
122.130
119.239
Br
O
O
O
Br
f1 (ppm)

2-Oxo-2-(thiophen-2-yl)ethyl thiophene-2-carboxylate (2i)

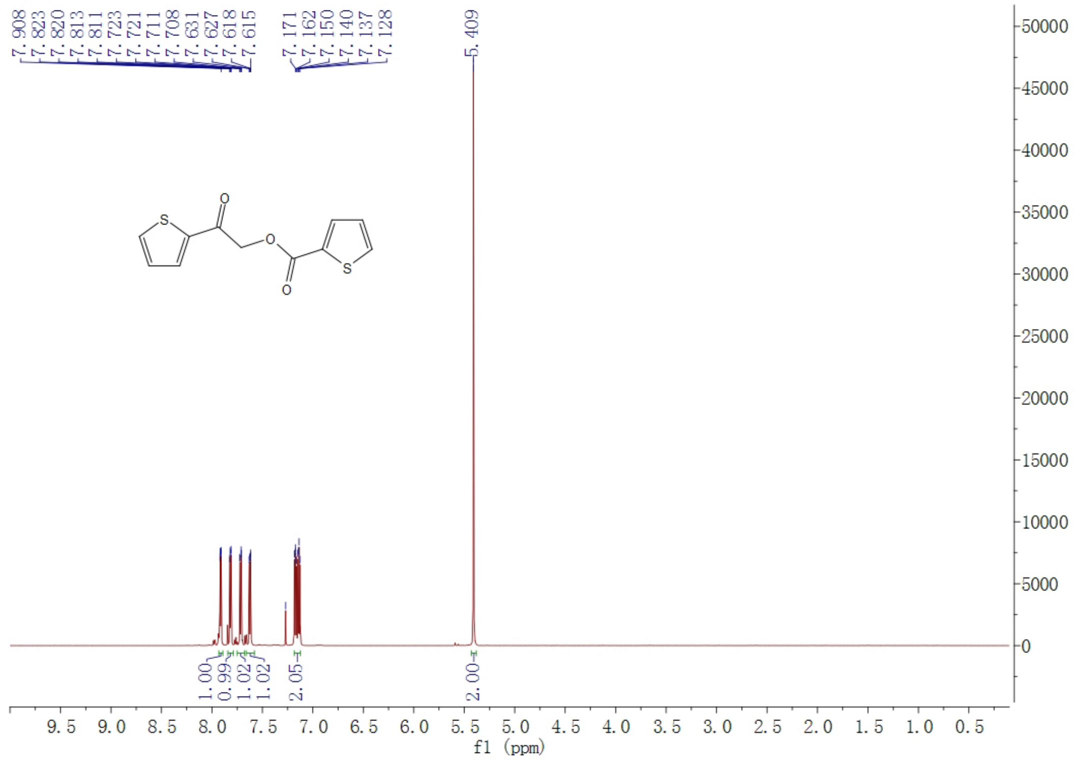

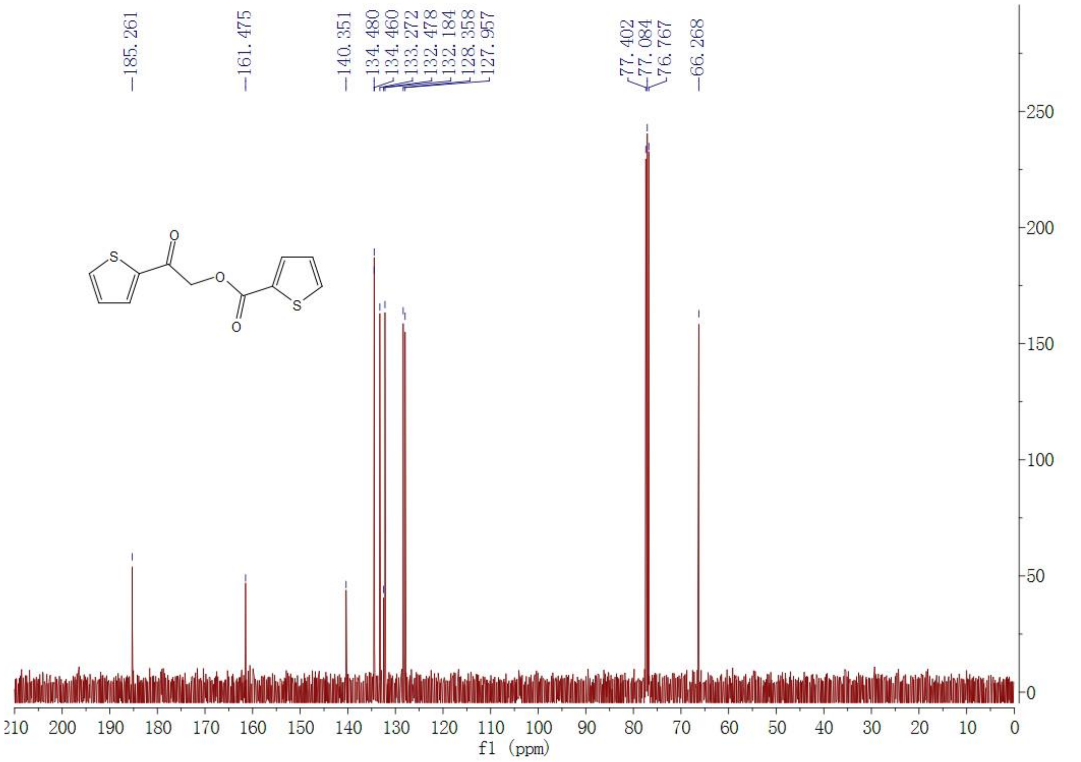

2-(Benzoyloxy)-1-phenyl-1-propanone (2j)

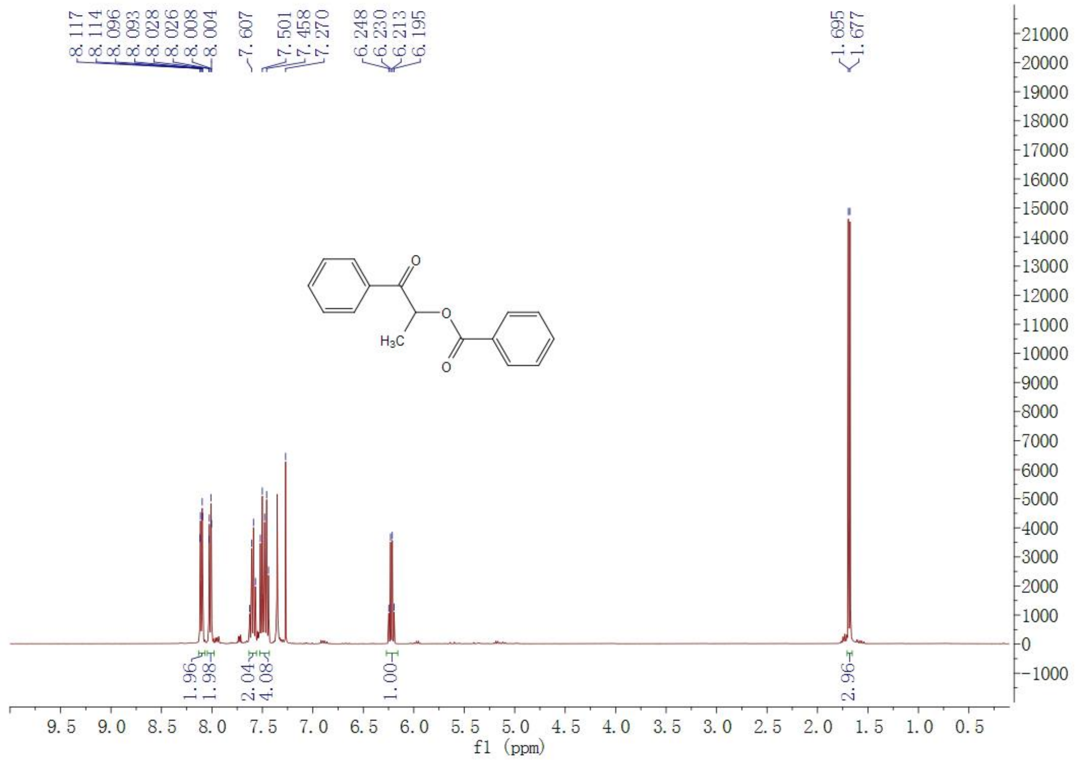

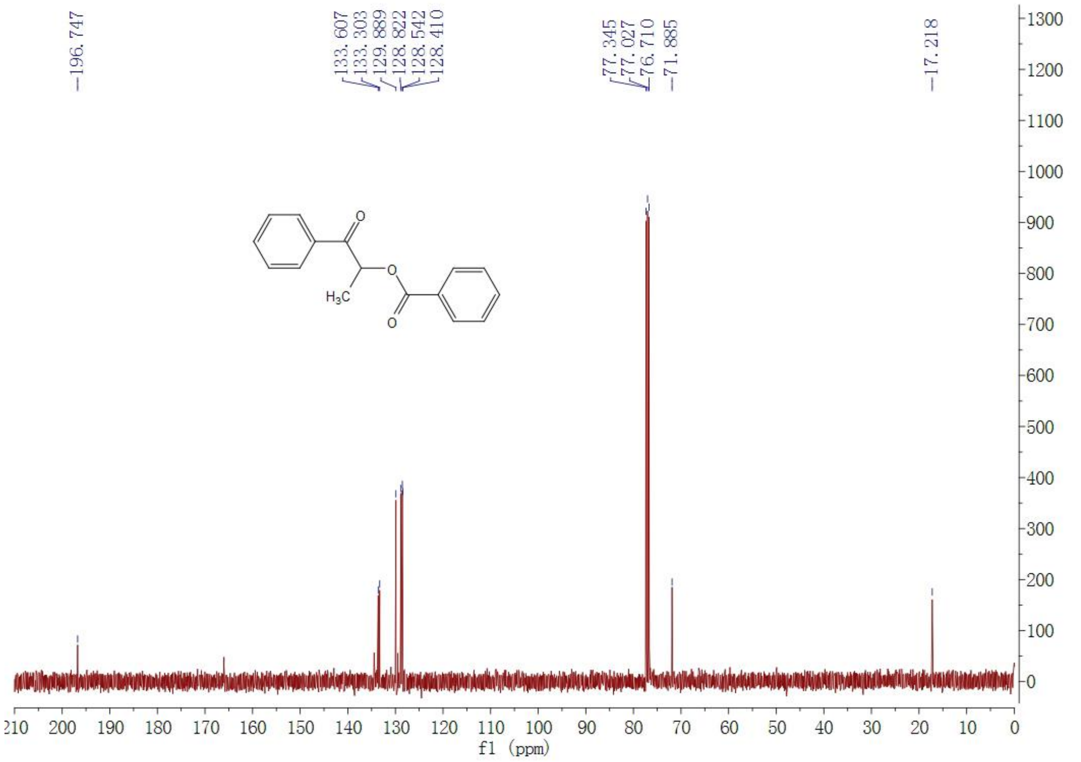

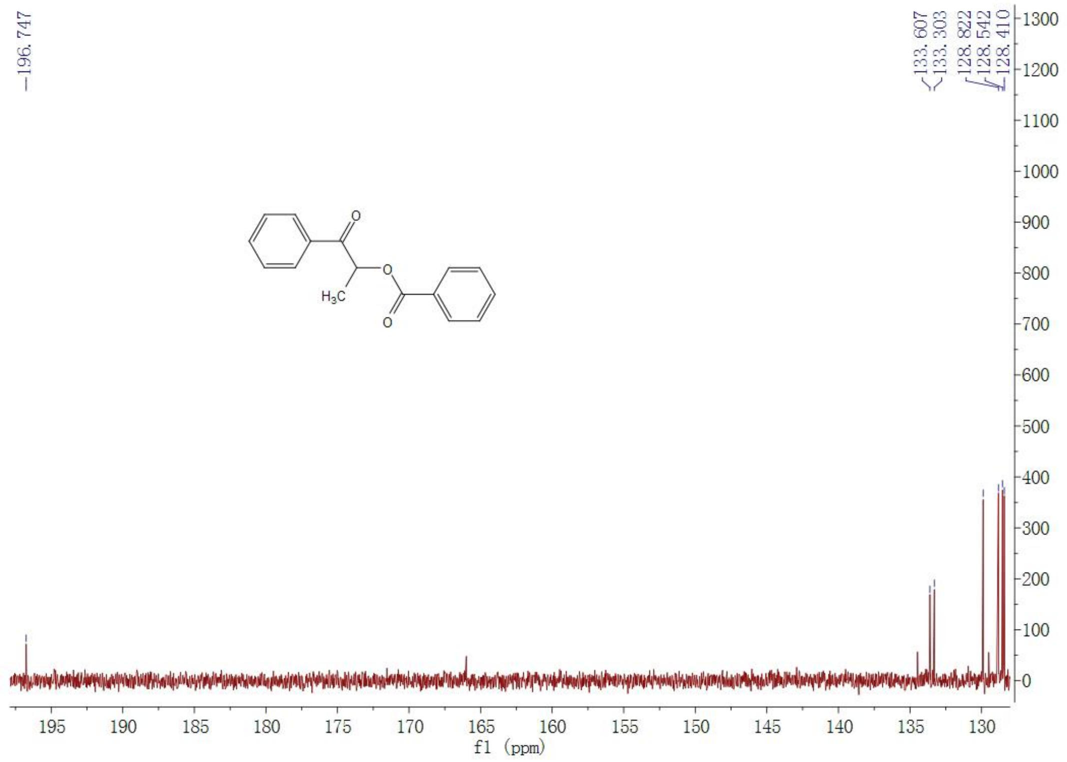

1-Oxo-1-p-tolylpropan-2-yl 4-methylbenzoate (2k) [4]

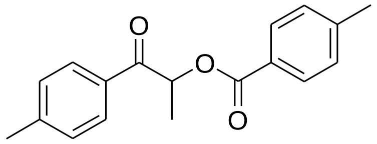

Yield: 76%; ^{1}H NMR ($CDCl_3$, 400 MHz) δ 8.00 (d, *J* = 8.0 Hz, 2H), 7.92 (d, *J* = 8.0 Hz, 2H), 7.30 (d, *J* = 8.0 Hz, 2H), 7.26 (d, *J* = 8.0 Hz, 2H), 6.19 (q, *J* = 7.2 Hz, 1H), 2.43 (s, 3H), 1.67 (d, *J* = 7.2 Hz, 3H); ^{13}C NMR ($CDCl_3$, 100 MHz) δ 196.4, 166.0, 144.4, 143.9, 131.9, 129.9, 129.4, 129.1, 128.6, 126.8, 71.6, 21.7, 17.2.

1-(4-Fluorophenyl)-1-oxopropan-2-yl 4-fluorobenzoate (2l)

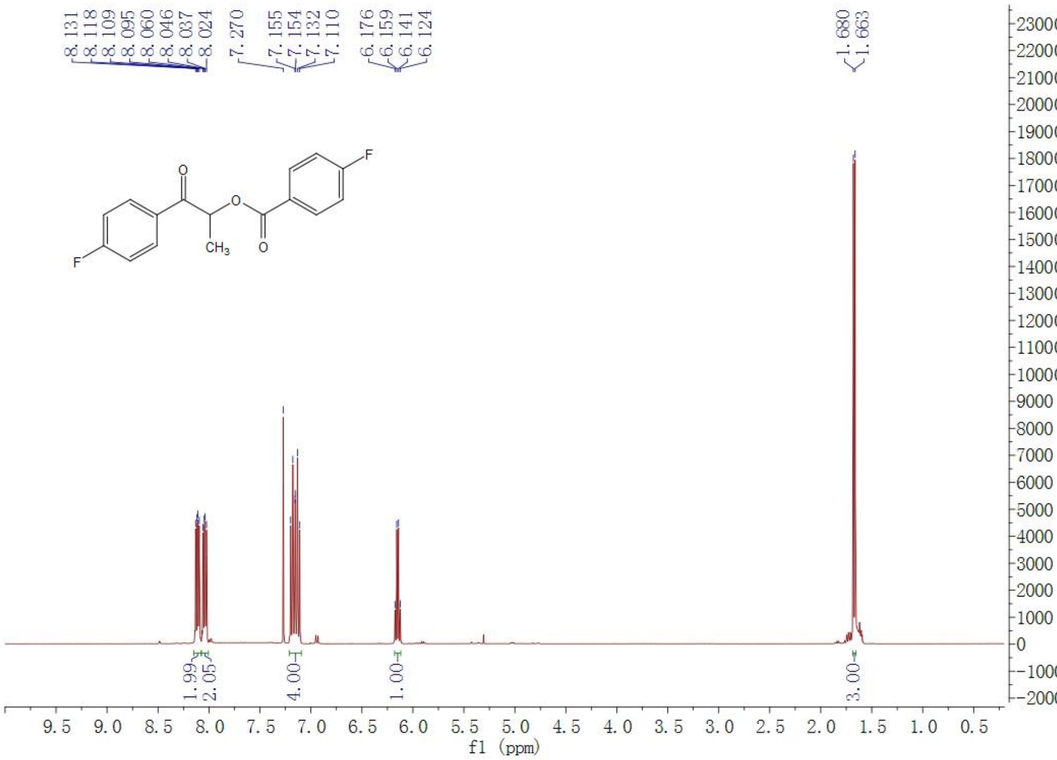

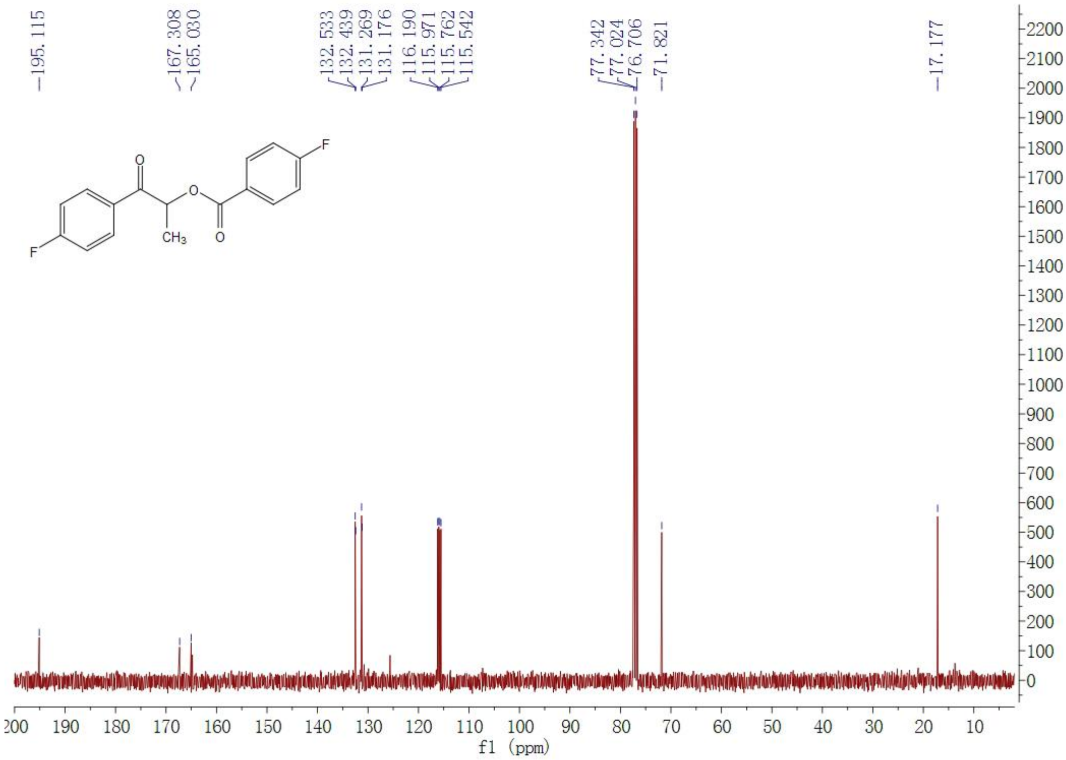

1-(4-Chlorophenyl)-1-oxopropan-2-yl 4-chlorobenzoate (2m)

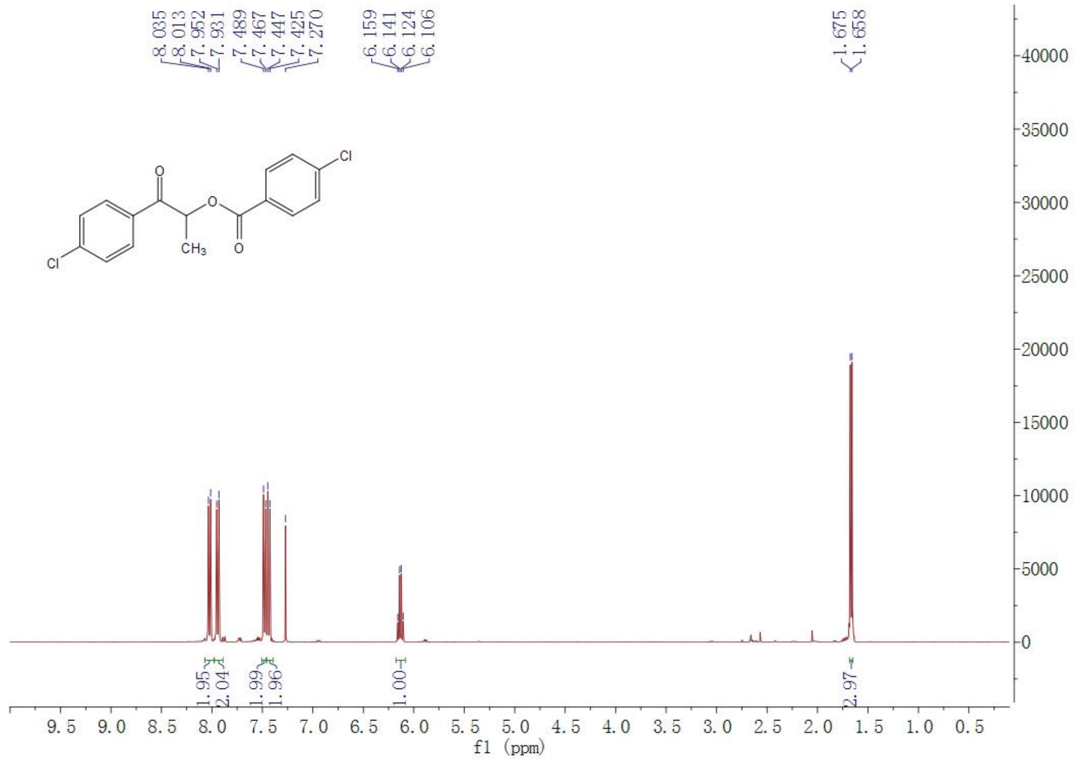

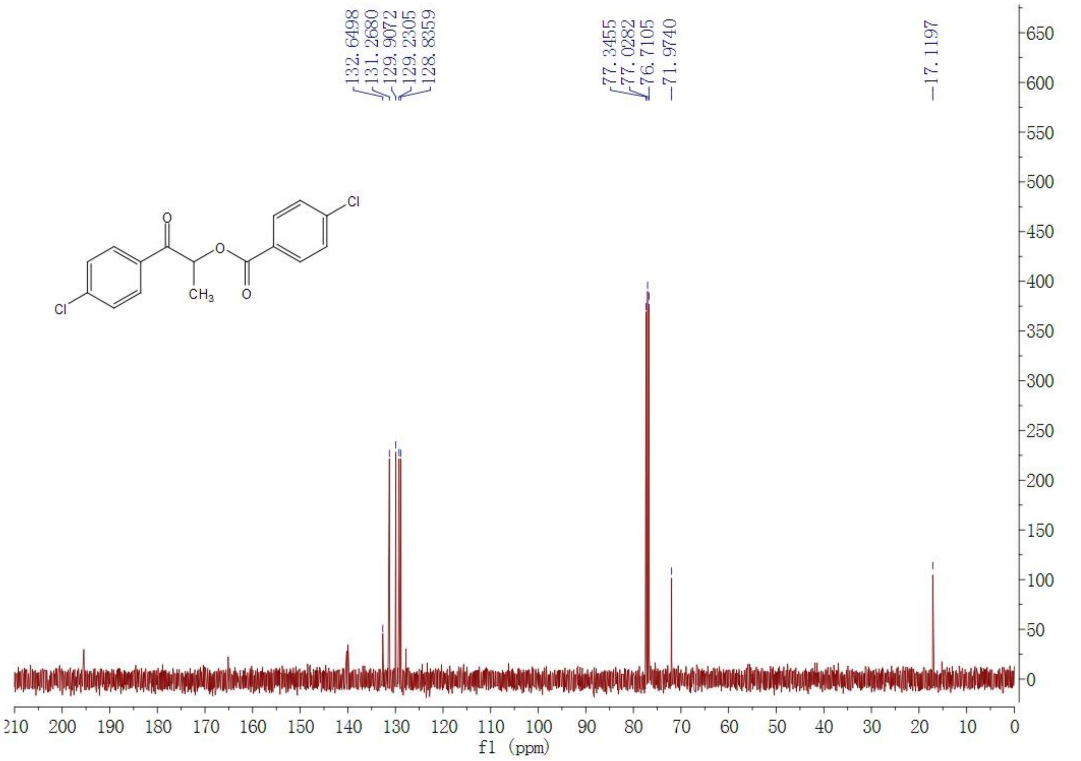

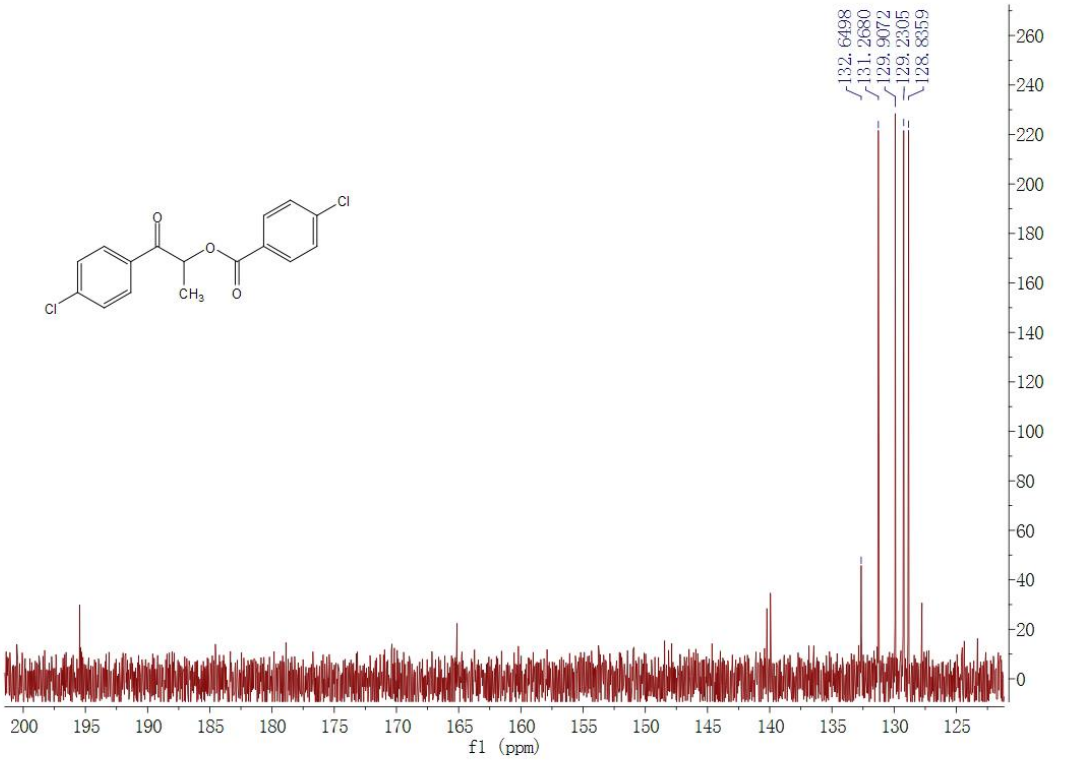

1-(4-Chlorophenyl)-1-oxobutan-2-yl 4-chlorobenzoate (2n)

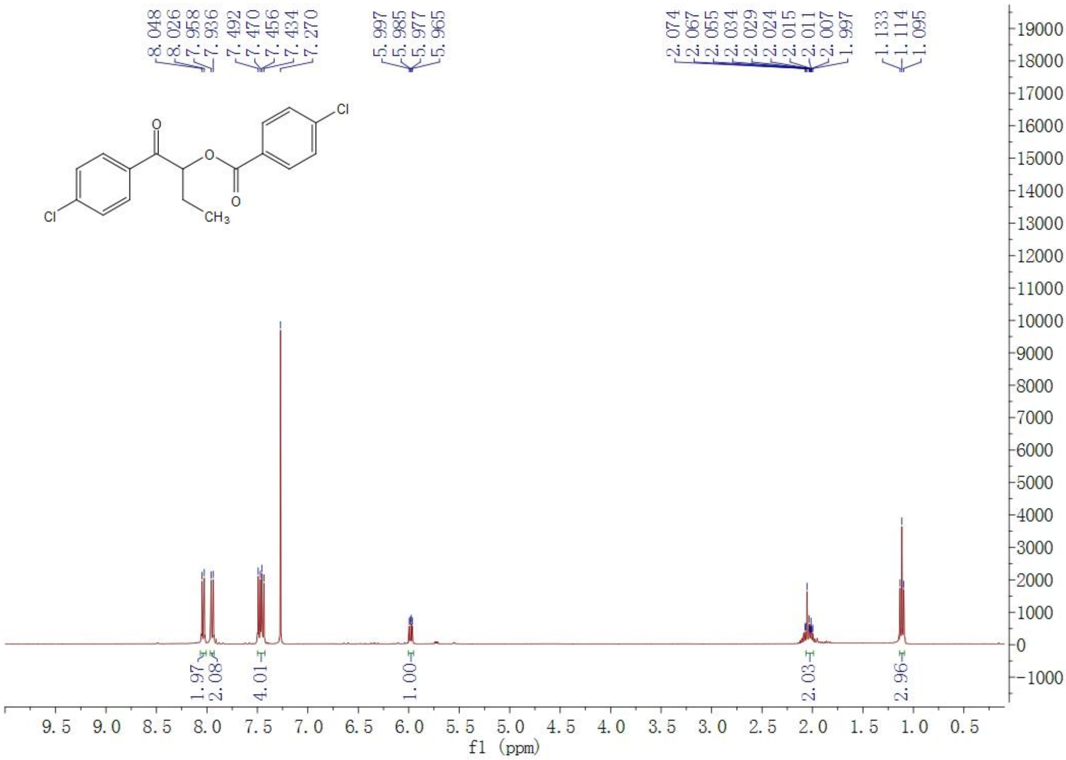

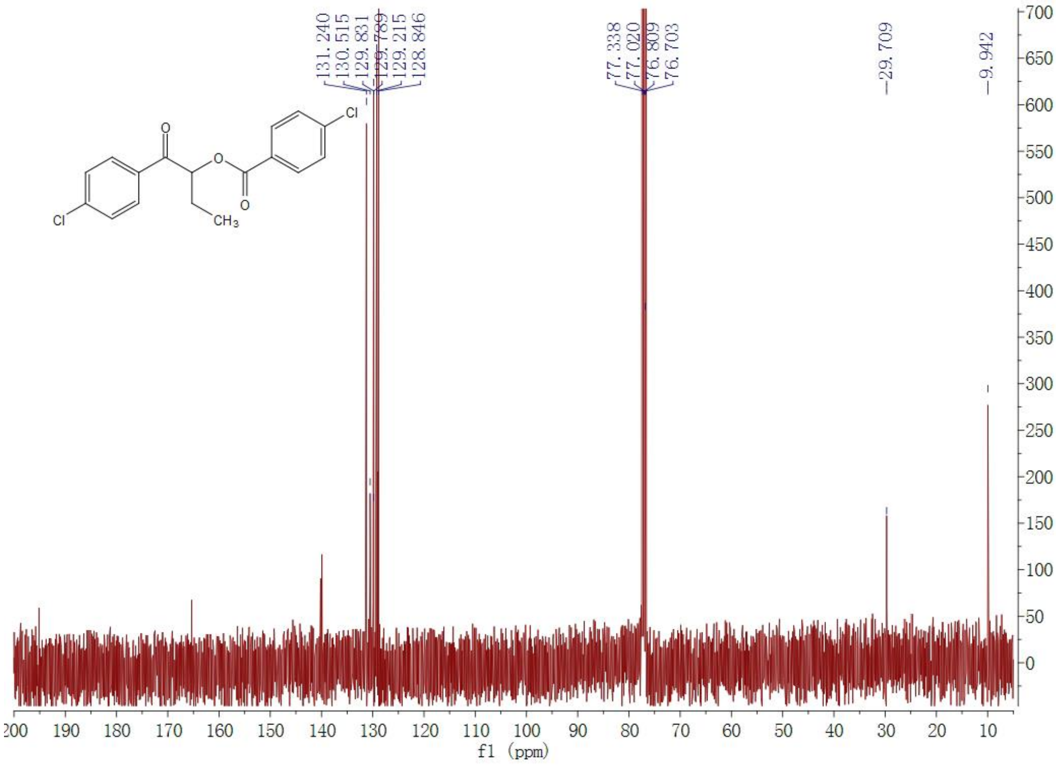
131.240
130.515
129.831
129.789
129.215
128.846
77.338
77.020
76.809
76.703
29.709
9.942
Cl
O
O
O
Cl
CH3
f1 (ppm)

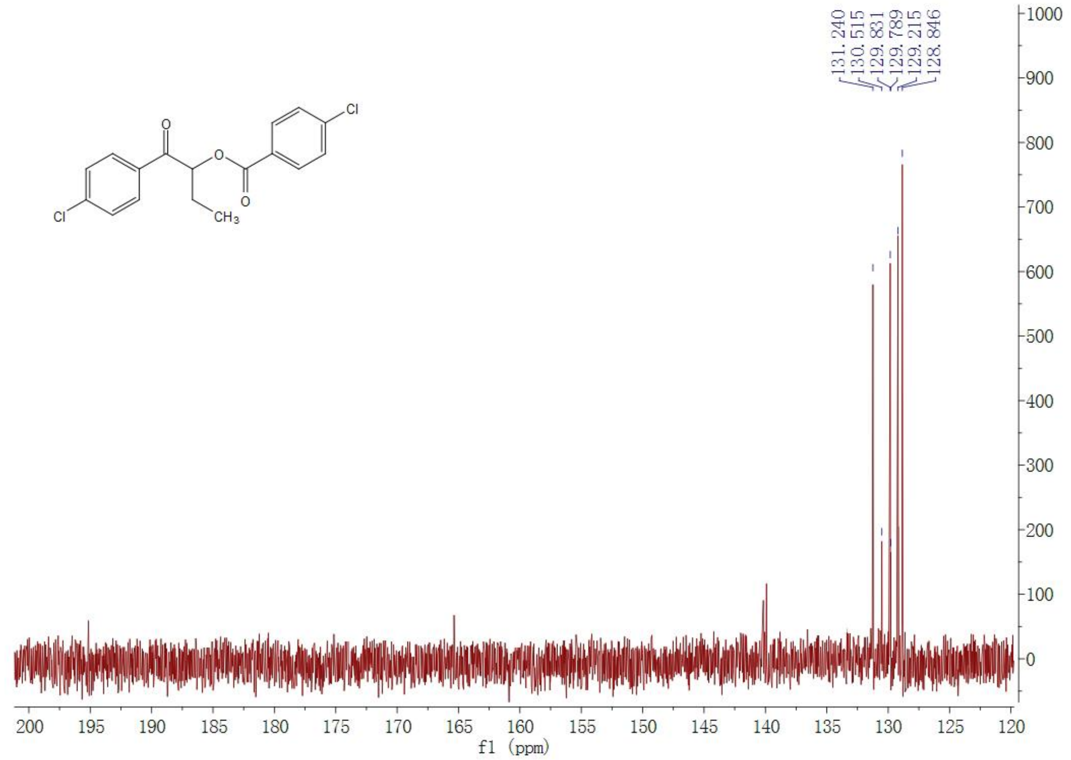
131.240
130.515
129.831
129.789
129.215
128.846
Cl
O
O
O
Cl
CH3
f1 (ppm)

References

[1] Ji, K. G.; Zhao, Y. L.; Zhang, L. M. *Angew. Chem. Int. Ed.* **2013**, *52*, 6508–6512.

[2] Khamarui, S.; Maiti, R.; Maiti, D. K. *Chem. Commun.* **2015**, *51,* 384–387.

[3] Zhu, M. H.; Wei, W.; Yang, D. S.; Cui, G.; Cui, H. H.; Sun, X. J.; Wang, H. *Org. Biomol. Chem.* **2016**, *14*, 10998–11001.

[4] Jia, W. G.; Zhang, H.; Li, D. D.; Yan, L. Q. *RSC Adv.* **2016**, *6*, 27590-27593.
